# Immunization targeting diseased proteins in synucleinopathy and tauopathy: insights from clinical trials

**DOI:** 10.1186/s40035-025-00490-9

**Published:** 2025-07-01

**Authors:** Xiaoni Zhan, Gehua Wen, Xu Wu, Jia-Yi Li

**Affiliations:** 1 School of Forensic Medicine, China Medical University, Shenyang, 110122 China; 2 Neural Plasticity and Repair Unit, Department of Experimental Medical Science, Wallenberg Neuroscience Center, Lund University, BMC A10, 22184 Lund, Sweden; 3 Health Sciences Institute, Key Laboratory of Major Chronic Diseases of Nervous System of Liaoning Province, China Medical University, Shenyang, 110122 China

**Keywords:** Passive immunotherapy, Tauopathy, Synucleinopathy, Tau, α‑Synuclein, Co-pathology

## Abstract

Synucleinopathies and tauopathies are neurodegenerative disorders characterized by the pathological accumulation of α-synuclein (α-syn) and tau proteins, respectively. These disorders are traditionally managed with symptomatic treatments without addressing the underlying pathologies. Recent advancements in passive immunotherapies, notably the FDA approval of the amyloid-beta (Aβ)-targeting antibody lecanemab, have sparked new hope in directly targeting pathological proteins. However, unlike the extracellular Aβ pathology, immunotherapies aimed at α-syn and tau, which predominantly form intracellular inclusions, face substantial challenges. To date, the therapeutic efficacy of five α-syn and 14 tau antibodies has been assessed in patients with synucleinopathies and tauopathies. These immunizations have demonstrated promising preclinical outcomes in alleviating pathological and behavioral deficits, but have not yielded significant clinical improvements in symptoms or measurable biomarkers. Therefore, a clear understanding of potential causes for the discrepancies between preclinical successes and clinical outcomes is critical for the successful translation of immunotherapy in the future. In this review, we examine existing passive immunotherapeutic strategies targeting α-syn and tau, specifically in patients with Alzheimer’s disease and Parkinson’s disease. Lessons learned from initial trial failures are also discussed, including refinement of animal models, inclusion and stratification of participants, improvement of clinical evaluations, and development of biomarkers. Given the overlapping pathologies and clinical manifestations of synucleinopathies and tauopathies, we further explore the potential of combined therapies targeting co-pathologies, offering novel insights for future therapeutic development against these neurodegenerative disorders.

## Introduction

After decades of effort and attrition in Alzheimer’s disease (AD) clinical trials, the recent FDA approval of lecanemab, a monoclonal antibody targeting amyloid-beta (Aβ), marks a significant breakthrough in passive immunotherapies targeting diseased proteins [1]. However, little progress has been made in clinical evaluations of immunotherapies targeting tau and α-synuclein (α-syn), key pathological proteins involved in tauopathies and synucleinopathies. Recently, the phase II PASADENA trial of prasinezumab, an antibody targeting aggregated α-syn, has indicated potential benefits in slowing motor progression among rapidly progressing subpopulations of Parkinson’s disease (PD) patients despite not meeting its primary endpoint [2, 3]. Concurrently, advancements in biomarker strategies have revolutionized the design of tau-targeting therapeutics, shifting the focus towards surrogate endpoints and exploring combination therapies targeting Aβ and tau [4–6]. In this review, we aim to synthesize and discuss the latest insights from preclinical and clinical trials on passive immunotherapy targeting α-syn and tau. We start with an overview of the physiological and pathological roles of both proteins to establish the foundation of their immunotherapy, with a special focus on their co-pathology in neurodegenerative diseases. Subsequently, we address the continuity and discrepancies between preclinical findings and clinical outcomes by reviewing published trials. Lastly, by analyzing potential factors that influence trial outcomes, we propose strategies and outline future perspectives to realize successful translation of immunotherapy for tau and α-syn.

## Key pathologies in tauopathy and synucleinopathies

### α-Syn and synucleinopathies

Synucleinopathies are a group of neurodegenerative disorders characterized by abnormal accumulation of misfolded α-syn. α-Syn forms intraneuronal inclusions known as Lewy bodies (LBs) and Lewy neurites (LNs) in PD, PD with dementia (PDD), and dementia with LBs (DLB). It is also present as glial cytoplasmic inclusions in oligodendrocytes in multiple system atrophy (MSA). Encoded by the *SNCA* gene, α-syn is a synaptic protein that regulates neurotransmitter release, synaptic function, and various cellular processes, including neuroplasticity, SNARE complex assembly, and vesicle trafficking [7]. Although predominantly located in the presynaptic terminals, approximately 5% of α-syn is typically released into the cerebrospinal fluid (CSF) and interstitial fluid (ISF) [8].

Structurally, the 140-amino-acid α-syn protein has three major domains that determine its physiological properties: the N-terminal amphipathic domain (residues 1–60), the central non-β-amyloid component (NAC) region (residues 61–95), and the negatively charged C-terminal domain (residues 96–140). The N-terminal amphipathic domain forms an α-helical structure essential for interaction with lipid membranes [9]. Familial PD mutations (e.g., A30P, E46K, H50Q, G51D, A53T) located in the N-terminal are associated with changes in the ability of α-syn to oligomerize [10]. The central NAC region is essential for aggregation. Misfolding of the NAC leads to formation of insoluble pathological forms of α-syn observed in neurodegenerative disorders [11]. The negatively charged C-terminal domain plays a role in maintaining the native protein state. Mutations or truncations within the C-terminus reduce negative charges and accelerate fibril formation [12, 13]. Additionally, the calcium-binding domain enhances the lipid-binding capacity of α-syn and facilitates its interaction with synaptic vesicles. Dysregulation of this domain is linked to vesicle clustering [14].

Post-translational modifications (PTMs) of α-syn, including ubiquitination, phosphorylation, oxidation, acetylation, O-GlcNAcylation, SUMOylation, nitration, and truncation, play crucial roles in LB and LN pathology [15–18]. As the most widely investigated site of phosphorylation, serine 129 (pS129) is associated with increased α-syn aggregation and toxicity. It significantly influences neuronal activity and enhances the interaction of α-syn with other critical synaptic proteins, such as VAMP2 and synapsin [19, 20]. However, a recent study suggests that hyperphosphorylation may prevent further aggregation, indicating a potential protective mechanism [21]. Additionally, α-syn truncation, particularly at the C-terminus, contributes significantly to the progression of synucleinopathies. Various forms of C-terminally truncated α-syn are present in both normal brains and LB disease brains [22–24], and are associated with increased aggregation propensity and decreased cell viability [25]. Transgenic mice expressing C-terminally truncated species manifest PD-like symptoms [26]. Nuclear magnetic resonance (NMR) spectroscopy evidence has revealed that the C-terminus interferes with the ability of the N-terminus to bind membranes and chaperones. Deleting C-terminal residues accelerates aggregation up to residues 85–90 located in the NAC domain [27, 28], providing a molecular basis for understanding the pathological role of C-terminal truncation in PD pathogenesis.

Under pathological conditions, intracellular α-syn monomers are prone to self-assemble into β-sheet-rich structures, forming oligomers and fibrils that ultimately become insoluble LBs. Oligomers are considered the most toxic species that cause neurotoxicity and play a critical role in PD pathophysiology [29, 30]. Advanced ligation assays and sensitive epitope unmasking techniques have shown that oligomers are more widespread in PD and DLB than previously recognized [31]. The toxicity is further exacerbated by their ability to seed intracellular aggregation in a prion-like manner—a process known as conformational templating, leading to spread through cell-to-cell transmission. This phenomenon has been observed in grafted cells transplanted into the brains of PD patients [32–34] and various animal models administered with preformed fibrils (PFFs) of α-syn [35, 36]. Furthermore, the propagation of pathological α-syn is enhanced by its templating-competent forms in the CSF of idiopathic PD subjects. Seed amplification assays (SAA) have revealed varying seeding capacities of CSF and plasma α-syn among diseases, offering a valuable diagnostic approach [37, 38]. α-Syn pathology can also be detected in peripheral organs such as the gut and skin during early stages of PD and MSA, providing a less invasive approach for diagnosis [39–42]. In addition, different α-syn strains have been extracted from the brains of patients with PD versus MSA, which exhibit structurally variable properties, as well as differential toxicity and seeding capabilities, reflecting different disease progression and clinical outcomes [43].

### Tau and tauopathies

Tauopathies encompass neurodegenerative disorders characterized by the accumulation of hyperphosphorylated tau proteins, which form neurofibrillary tangles (NFTs) within neurons or glial cells [44]. Tauopathies are categorized into primary and secondary tauopathies. Primary tauopathies are characterized by tau protein aggregate as the predominant pathology, including frontotemporal dementia, Pick’s disease, progressive supranuclear palsy (PSP), and corticobasal degeneration (CBD). In contrast, secondary tauopathies, such as AD, involve a mixed pathology that includes both NFTs and Aβ plaques [44, 45].

Tau, encoded by the *MAPT* gene on chromosome 17q21-22, is a microtubule-associated protein predominantly located in neurons. It plays a crucial role in promoting microtubule assembly and stability, facilitating axonal transport, and supporting neurite outgrowth. A small amount of tau is also present in dendrites, where it mediates excitotoxicity and regulates synaptic plasticity, post-synaptic microtubule dynamics, and neuronal overall physiology [46]. Tau has also been detected extracellularly, contributing to pathology spreading via intercellular transmission [47, 48]. Extracellular tau aggregates exhibit varied isoforms and phosphorylation states. They influence synaptic connectivity and mitochondrial responses, leading to synaptic transmission dysfunction in neurons and impairing astrocytes’ ability to clear extracellular glutamate [49, 50]. Extracellular tau oligomers lead to neuronal nuclear invagination, impairing nucleocytoplasmic transport, which may exacerbate neurodegenerative diseases [51].

Structurally, tau comprises four primary domains: an N-terminal projection domain, a proline-rich region, a microtubule-binding repeat (MTBR) domain, and a C-terminal region. Alternative splicing of *MAPT* produces six isoforms that differ in the presence of N-terminal inserts (0N, 1N, 2N) and the number of MTBR domains (R1, R2, R3, and R4). Dimerization of tau is initiated through interactions between hexapeptide motifs VQIINK and VQIVYK located in the R2 and R3, respectively. This dimerization is crucial for the nucleation and elongation processes that lead to tau aggregation [44, 52]. Ultimately, imbalanced expressions of 3R and 4R tau are crucial in tauopathy pathogenesis. Depending on the predominant tau isoform that accumulates, tauopathies can be categorized as 3R tauopathies (Pick’s disease), 4R tauopathies (PSP), and mixed 3R/4R tauopathies (most of the secondary tauopathies).

Pathologically, tau undergoes various PTMs [53]. High levels of ubiquitinated tau have been found in AD and other tauopathies. Tau ubiquitination, along with lysine acetylation, impairs tau function and promotes its aggregation [53, 54]. Hyperphosphorylation is the most extensively studied and prominent pathological feature of tau. This modification decreases the affinity of tau to microtubules, facilitating the transformation of soluble tau into insoluble oligomers and paired helical filaments (PHFs) [53, 55]. This process is also associated with synaptic disruptions and impaired mitochondrial trafficking, contributing to neurodegeneration [53, 56, 57]. Remarkably, phosphorylated tau (p-tau) forms in MTBR, such as p-tau181, p-tau217, and p-tau231, are elevated in the CSF and blood of AD patients, underscoring their utility as biomarkers for diagnosis and intervention assessment. A recent study suggests that p-tau217 is superior to p-tau181 for the differential diagnosis of AD [58–60]. Additionally, p-tau243 is particularly specific to tau aggregates in late-stage AD and is strongly associated with tau positron emission tomography (PET) imaging [61]. Furthermore, tau truncation promotes aggregation of tau protein, thus playing a potential role in disease progression [44, 53, 62]. Both N- and C-terminally truncated tau fragments exert pathological effects. The C-terminal fragments impair the microtubule function, and enhance the prion-like propagation and aggregation of tau [44, 53, 63]. Fragments derived from N-terminal tau truncation secreted into the CSF have synaptotoxic actions, and their phosphorylation is a major source of biomarkers [64]. The truncated tau fragment (1–368) notably enhances *BACE1* (beta-secretase 1) expression and Aβ production, further propagating AD pathology [65].

Tau is present in various forms, ranging from soluble monomers to insoluble fibrils. Insoluble NFTs are classic toxic entities that correlate with the duration and severity of neurodegenerative diseases [44, 66]. However, there is an ongoing debate on whether tau aggregation results from a toxic gain of function or a loss of normal function. Like α-syn, tau oligomers are considered more toxic than fibrils due to their capacity to impair memory consolidation by inducing synaptic and mitochondrial dysfunction [67]. Additionally, tau oligomers can damage neuronal nuclei, impairing nucleocytoplasmic transport and altering pathogenic gene expression [51]. Tau fibrils exhibit prion-like properties, which encompass seeding and promoting fibril formation in normal tau proteins, as well as to facilitate the inter-neuronal spread of tau aggregates [68]. Structural studies using cryo-electron microscopy have uncovered different tau strains in various tauopathies, such as Pick’s disease, chronic traumatic encephalopathy (CTE), CBD, and PSP. These strain-specific properties are preserved upon seeding, highlighting the structural heterogeneity of tau in different disorders [69, 70]. This heterogeneity significantly influences the efficacy of therapies targeting tau fibrils.

### Co-pathology of α-syn and tau

The co-presence of α-syn and tau is commonly observed in synucleinopathies and tauopathies [71–73]. Notably, intracellular α-syn deposition is found in over 50% of AD patients [73–75], while p-tau has been spotted in LBs in the medulla of 80% of AD and diffuse LB disease cases [76]. Recent studies indicate that CSF α-syn may influence CSF total tau and p-tau181 levels through inflammatory pathways involving TNFR1 (tumor necrosis factor receptor 1) and ICAM-1 (intercellular cell adhesion molecule-1). Higher CSF α-syn levels are associated with cognitive decline among non-demented aged subjects [77]. Furthermore, antibodies for tau PHFs can partially label LBs within the same neuronal cells in brains of familial PD and DLB cases, suggesting a pathological continuum between these diseases [78]. Several genome-wide association studies (GWAS) have provided genetic evidence for their overlaps [79, 80]. Meta-analysis studies have repeatedly spotted the *MAPT* H1 haplotype as a risk locus for PD onset [81]. Meanwhile, multiple *MAPT* inversion polymorphisms have been found to increase the risk of PD and are strongly associated with the development of dementia among PD patients [82].

Molecular interactions between the two proteins have been elucidated in vitro. Evidence from NMR spectroscopy and cryo-EM shows that α-syn directly interacts with the MTBR of tau through its C-terminus, synergistically promoting amyloid fibrillation and co-aggregation [83–85]. The α-syn NAC (71–82 aa) can interact with two hexapeptide motifs of tau (^275^VQIINK^280^ and ^306^VQIVYK^311^) through hydrophobic forces [86]. Tau and α-syn can also form electrostatic complex coacervates and co-aggregate through liquid–liquid phase separation (LLPS), where the C-terminal region of α-syn interacts with the P2 proline-rich region of tau, providing a biophysical explanation for co-pathology formation [87, 88]. Notably, different strains of α-syn exhibit varying abilities to induce tau aggregation, significantly impacting their cross-seeding abilities [89–91]. New strains formed from the interplay between the two proteins display enhanced neurotoxicity and stronger propagation ability [90, 92, 93]. An in vivo study demonstrated that administration of combined α-syn/tau oligomers derived from PD patients accelerates tau oligomer formation and induces more severe neuronal loss in tau transgenic mice compared to administration of tau oligomers alone [90]. Similarly, administration of a mixture of AD lysate-derived tau and α-syn fibrils significantly increases p-tau aggregation and exacerbates the spread of tau pathology. Notably, the absence of endogenous α-syn in mice reduces the accumulation and spreading of tau but does not affect the seeding or spreading capacity of α-syn [94]. In addition, unilateral hippocampal injection of tau/α-syn co-polymers exacerbates the neuroanatomic transmission of seeded tau pathology compared to tau fibril alone, but does not trigger endogenous α-syn pathology. This suggests that co-polymers possess more potent transmission properties, preferentially in tauopathy models rather than in synucleinopathy [95].

Recently, a study proved that the α-syn and tau co-pathology can spread from the gut to the brain and subsequently propagate into the dorsal motor nucleus of the vagus (DMV) or the nucleus of the solitary tract (NTS) and other brain regions in gut-inducible transgenic mouse models, where the propagation was significantly inhibited by truncal vagotomy and α-syn deficiency [96]. These findings highlight a more pronounced effect of α-syn amyloidogenicity than tau, reflecting a potential directional cross-seeding effect of α-syn on tau, but not vice versa. Remarkably, the distinct distribution patterns of α-syn/tau co-pathology in various disorders — co-pathology in the limbic and olfactory regions in AD versus co-pathology in the substantia nigra and brainstem in PD — highlight their heterogeneity and present challenges for differential diagnosis and development of targeted immunotherapies [72, 73].

## Current immunotherapies for synucleinopathies and tauopathies

### Rationale for passive immunization targeting diseased proteins

Passive immunization involves administration of preformed antibodies to provide immediate protection. This approach allows for direct control over antibody titers and typically exhibits fewer side effects, making it safer than active immunization—particularly for the elderly individuals, as well as AD and PD patients, who often exhibit reduced vaccine responsiveness [97, 98]. Although the causality of the two proteins for the manifestation of synucleinopathy and tauopathy remains unclear, both genetic and pathological evidence supports the rationale for targeting α-syn and tau through immunotherapy.

Clinical data indicate that the *SNCA* gene expression is dose-dependently linked to the severity of synucleinopathy. Cases with *SNCA* duplication show milder forms of late-onset PD, while *SNCA* triplications result in more complex clinical manifestations, including parkinsonism, autonomic dysfunction, and dementia [99, 100]. Additionally, GWAS has identified numerous genetic variants of *SNCA* as a risk factor. Individuals with missense mutations at the *SNCA* locus develop autosomal-dominant PD [99, 101, 102]. Over 80 *MAPT* mutations have been discovered to be associated with tauopathies [44, 103]. For instance, P301L mutation is a common cause of FTDP-17 (frontotemporal dementia and Parkinsonism linked to chromosome 17) [104]. *MAPT* IVS10 + 16 mutation affects exon 10 splicing, favoring production of the 4R tau isoform and resulting in phenotypes similar to PSP or CBD [105]. In transgenic mouse models carrying the R406W mutation, hyperphosphorylated tau inclusions and AD-like associative memory deficits have been observed, albeit without Aβ deposition [106]. Moreover, tau filaments in individuals with R406W and V337M mutations adopt the same structural conformation as those seen in the brains of AD patients [107]. Notably, these phenomena have been accurately depicted in cells and transgenic mouse models expressing *SNCA* or *MAPT* mutations. These models show progressive accumulation of α-syn or tau aggregates, accompanied by subsequent neurodegenerative phenotypes. Genetic deletion of α-syn or tau in these models alleviates the symptoms of synucleinopathy and tauopathy [108, 109]**.**

Moreover, in rodents and nonhuman primates, infusions of brain extracts or α-syn or tau PFFs precipitate the onset of endogenous α-syn or tau accumulation, leading to their widespread propagation within the brain [35, 110–113]. Notably, recent studies have discovered that the link between tau pathology and neuronal damage is more robust than that between amyloid plaques and neurodegeneration [114, 115]. While tau deposition in the temporal lobe is more predictive of dementia status and cognitive performance, tau deposition in the entorhinal cortex is linked to earlier symptom onset [116, 117]. Furthermore, tau abnormalities can precede amyloid plaques and may drive AD progression by exacerbating amyloid pathology [118]. Tau mediates Aβ toxicity through postsynaptic targeting of the Src kinase Fyn, which leads to neuronal damage in AD models [44, 119].

These lines of evidence highlight the potential of tau-targeting immunotherapy for AD. Unlike the early Aβ-targeting treatment, passive immunization targeting α-syn and tau generally show few complications. Nevertheless, it is more complicated as both α-syn and tau are mainly located intracellularly. The primary goal of both strategies is to target extracellular proteins, thereby blocking seeding, preventing cell-to-cell propagation, and inhibiting aggregation. Encouragingly, some pre-clinical studies have provided promising results of antibody internalization through membrane-mediated endocytosis in neurons, thereby reducing intracellular proteins. Additionally, glial cells contribute to the clearance of the monoclonal antibody-protein complex, enhancing the therapeutic impact.

Passive immunotherapy offers several advantages over other therapeutic strategies targeting α-syn and tau, such as antisense oligonucleotides (ASOs) [120, 121], kinase inhibitors [122], and proteolysis-targeting chimeras (PROTACs) [123, 124]. The antibodies are highly selective to specific pathological species (e.g., oligomers or fibrils), largely reducing off-target effects. In contrast, ASOs broadly affect total protein levels, while kinase inhibitors and PROTACs impact multiple substrates and pathways in proteasomal degradation. Moreover, the prolonged half-life of monoclonal antibodies (ranging from weeks to months) contrasts with the rapid metabolism of PROTACs and ASOs, allowing less frequent administration and better evaluation of pharmacokinetics and pharmacodynamics. Moreover, unlike PROTACs and ASOs, passive immunotherapies targeting extracellular α-syn and tau avoid innate immune responses or cellular stress. Nevertheless, the antibodies have limited blood–brain barrier (BBB) penetration, which largely restricts their efficacy against intracellular pathology [125]. In addition, compared to small-molecule inhibitors, passive immunotherapy is associated with higher cost and greater complexity of treatment administration, leading to variability in efficacy across patients. Overall, passive immunotherapy is a safe and promising approach for clinical application, but alternative strategies are essential for enhancing the treatment efficacy.

### Current clinical trials of anti-α-syn immunotherapy

Currently, five antibodies targeting α-syn have entered phase II clinical trials in patients with PD and MSA (Table 1). Among these, only cinpanemab targets the N-terminus of α-syn, while the other four target the C-terminus. A pivotal aspect of α-syn passive immunization is the specific targeting of pathogenic oligomeric and aggregated forms. This strategy, employed in the development of therapeutics such as cinpanemab, prasinezumab, and ABBV-0805, aims to reduce the levels of misfolded protein, halt the spread of pathogenic α-syn aggregates, and potentially modify disease progression while preserving physiological functions. However, despite the efficacy of target engagement in plasma and CSF, no significant improvements in clinical symptoms were observed after one to two years of treatment. Encouragingly, recent subgroup analyses in the phase II clinical trials of prasinezumab and Lu AF82422—conducted in PD and MSA patients, respectively—have yielded promising results [3].

**Table 1:** Clinical studies of antibodies targeting α-syn and Tau

		Name	Origin/development	Fc region (IgG subclass)/binding epitope (aa)/target αSyn form	Clinical relevance	Abbreviation and NCT number/status/start date and end date	Participants	Dose and duration of Phase II testing	Clinical symptom evaluation	Imaging monitoring; biomarker Screen	Refs.
Anti-αsyn mAb	N-terminal	Cinpanemab/BIIB054/NI-202	Fully human, Isolated from B cells; Biogen Int Neuroscience GmbH, Neurimmune	IgG1; N-Terminal aa 4–10 (FMKGLSK); Aggregated, fibrillar, preferably oxidized αSyn	No significant clinical improvement	SPARK; NCT03318523; Phase II (Terminated); 2018–01 to 2021–04	357 PD patients (< 3 years from diagnosis and H&Y < 2.5) DaT-SPECT positive	Year 1: 250 mg, 1250 mg, or 3500 mg i.v. Q4W Year 2: placebo randomized to active treatment	PE: MDS-UPDRS Total Score (52/72 w) SE: MDS-UPDRS Subpart Score (52/72/96 w); DaT-SPECT (52 w), antibody serum concentration; AEs and SAEs	DaT-SPECT; –	[129, 130]
	C-terminal	Lu AF82422	– H. Lundbeck A/S, Genmab A/S	IgG1; C-terminal; All major species (monomeric and oligomeric; N- or C-terminal truncated forms)	42% slowing on the modified UMSARS in the less impaired subgroup (post hoc study); Trends slowing on UMSARS Total and Subpart Score; Trends to slowing on regional MRI volumetric reduction (pons and cerebellum), and NfL in the CSF;	AMULET; NCT05104476 Phase II (Active, not recruiting,enter one-year OLE period); 2021–11 to 2026–05	64 MSA patients Parkinsonian and Cerebellar type	4200 mg i.v. Q4W (48 to 72 w) + OLE (up to week 92)	PE: UMSARS Part I and Part II Total Score SE: UMSARS Subpart Score. Symptoms, daily function, volumetric MRI, pharmacokinetics	MRI; NfL	–
					–	MASCOT; NCT06706622; Phase III (Recruiting, New); 2024–12 to 2029–10	360 MSA patients Parkinsonian and Cerebellar type	Low, high, and placebo i.v	PE: mUMSARS SE: UMSARS Subpart Score, CGI, pharmacokinetics, AEs and SAEs	– –	–
		MEDI1341/ TAK-341	Fully human; generated from lead isolate asyn0087; Astra Zeneca, Takeda Pharmaceutical Company, MedImmune (originator)	IgG1 with reduced effector function by triple mutation; C-terminus within aa 102–130; Aggregated, monomeric αSyn	–	NCT05526391; Phase II (Active, not recruiting); 2022–11 to 2025–07	138 MSA patients	Unknown i.v. Q4W to 52 w	PE: UMSARS-Part 1 (52 w) SE: UMSARS-Total1; CGI; PK, other clinical measures, survival time, AEs and SAEs, and antidrug antibodies	– –	–
		Prasinezumab/PRX002	Humanized version of murine 9E4 antibody; Prothena Biosciences Limited, Hoffman-LaRoche	IgG1; C-terminal around aa 122, suppose 118–126 (based on 9E4 antibody epitope mapping); Aggregated αSyn	Potential benefit on UPDRS-Part III, 40%–64% decline in subgroup with advanced symptoms (post hoc study); Worsening on the MDS-UPRDS part II and III significantly lagged behind after 3 ~ 4 years treatment	PASADENA; NCT03100149; Phase II (Active, not recruiting); 2017–06 to 2026–09	316 PD patients newly diagnosed and mild symptom	Part 1: 1500 mg, 4500 mg or placebo i.v. Q4W to 52 w Part 2: placebo change to 1500 mg for extra 52 w Part 3: 1500 mg i.v. Q4W for all participants for 260 w OLE	PE: MDS-UPDRS Total Score (52 w) SE: MDS-UPDRS Part IA, Part IB, Part I, Part II, Part III Total and Subscores; DaT-SPECT; MoCA Total Score; CGI-I etc	DaT-SPECT; –	[2, 3]
					–	PADOVA; NCT04777331; Phase IIb (Active); 2021–05 to 2026–11	575 early PD patients more advanced symptoms	1500 mg monthly i.v. (18 m) + 2 years OLE	PE: Time to confirmed motor progression event SE: MDS-UPDRS Part II; CGI; MDS-UPDRS Part III/ IV Additional measures of motor function, AEs and SAEs, pharmacokinetics, and anti-drug antibodies	– –	–
		Exidavnemab/ABBV-0805/ BAN0805	Humanized mAb47; BioArctic AB	IgG1/4 with possible modification to reduce complement component 1q (C1q) binding C-terminal aa 121–127 (based on mAb47 antibody epitope mapping); Preferably aggregated αSyn	–	EXIST; NCT06671938; Phase IIa (Recruiting, New); 2024–11 to 2026–03	24 PD patients mild to moderate symptomatic	Two unknown doses and placebo i.v. Q4W	PE: AEs and SAEs SE: Pharmacokinetics, and immunogenicity effects	– –	–
Anti-tau mAb	N-terminal	Gosuranemab/BIIB092	Isolated from familial AD patient-derived pluripotent stem cells; Biogen, Bristol-Myers Squibb	lgG4; N-terminal aa 15–22; tau monomer, fibrils, and insoluble tau	No significant clinical improvement	PASSPORT; NCT03068468; Phase II (Terminated); 2017–06 to 2020–02	490 PSP patients	1–48 w: 50 mg/mL and placebo i.v. Q4W 52–208 w: 50 mg/mL i.v. Q4W for all participants	PE: PSPRS (52 w); AEs and SAEs SE: MDS-UPDRS-Part II; CGI-C, etc. vMRI	vMRI; –	[174, 175]
					Reduced CSF unbound N-terminal tau fragments,total tau and p- tau unchanged; Worsening on the ADAS-Cog13 secondary endpoint with highest dose in 18 m	TANGO; NCT03352557; Phase II (Terminated); 2018–05 to 2021–08	654 MCI or mild AD patients (positive amyloid PET scan)	Low, medium, high dose i.v. Q4W	PE: AEs and SAEs SE: CDR-SB; anti-BIIB092 resistence	– –	[176]
		Tilavonemab/C2N-8E12	Humanized mAb derived from HJ8.5; AbbVie, C2N Diagnostics, LLC	lgG4; N-terminal aa 25–30; Soluble extracellular tau	No significant clinical improvement	NCT02880956; Phase II (Completed); 2017–01 to 2021–07	453 Early AD	300 mg, 1000 mg, 2000 mg and placebo i.v. Q4W for 96 w	PE: CDR-SB; AEs SE: Pharmacokinetics, ADAS-Cog-14	– –	[168]
					No significant clinical improvement besides decreases in CSF free tau and higher plasma total tau	ARISE; NCT02985879; Phase II (Terminated); 2016–12 to 2019–11	378 PSP patients	2000 mg, 4000 mg and placebo i.v. Q2W (first 3 injections), then i.v. Q4W for 52 w	PE: PSPRS Total Score; AEs SE: UPDRS-Part II; CGI-C; MRI, Pharmacokinetics. etc	MRI; –	[146]
		Semorinemab/RO7105705	Humanized variable regions of muMtau; AC Immune SA, Genentech, Hoffmann-La Roche	lgG4; N-terminal aa 6–23; Both monomeric and oligomeric tau, regardless of phosphorylation status	No significant clinical improvement besides decreases in CSF total tau and phosphorylated tau	TAURIEL; NCT03289143; Phase II (Completed); 2017–10 to 2021–01	457 Prodromal to mild AD (MMSE ≥ 20 and CDRGS 0.5 or 1)	1500 mg, 4500 mg, or 8100 mg and placebo i.v. Q2W (first 3 infusions) Q4W for 49–61 w + OLE to 96 w	PE: ADAS-Cog11; ADCS-ADL SE: CDR-SB, MMSE etc	[^18^F] GTP1 PET imaging; –	[156]
					43.6% slowing of decline on the ADAS-Cog11 co-primary (word recognition); Reductions in CSF total tau and p-tau181, biomarkers for neuroinflammation unchanged	LAURIET; NCT03828747; Phase II (Completed); 2019–01 to 2023–08	272 moderate AD patients (MMS of 16–21 and CDR-GS 1 or 2)	4500 mg and placebo i.v. Q2W (first 3 infusions) + Q4W in the OLE period	PE: ADAS-Cog11. ADCS-ADL SE: ADAS-Cog13 Score; ADCS-iADL Score; CDR-SB; MMSE; vMRI etc	[^18^F] GTP1 PET imaging/ MRI; –	[157]
	N-terminal to mid-domain	Zagotenemab	Humanized antibody derived from MCI-1; Eli Lilly & Co	lgG4; N-terminal aa 7–9, 312–322; Soluble tau aggregate	No significant clinical improvement	PERISCOPE-ALZ; NCT03518073; Phase II (Completed); 2021–04 to 2021–10	360 early symptomatic AD patients	1400 mg, 5600 mg and placebo i.v. Q4W (100 w)	PE: iADRS SE: ADAS-Cog13; ADCS-iADL; CDR-SB; MMSE	[^18^F] GTP1 PET imaging/ MRI; –	[179]
	Mid-domain	BIIB076	NI-105.6C5 of healthy human B cells; Biogen, Eisai Co., Ltd., Neurimmune	IgG1; Mid-domain aa 125–131; Monomeric and fibrillar tau	–	NCT03056729; Phase I (Completed); 2017–02 to 2020–03	24 healthy volunteers and AD patients	Single-escalating- dose i.v. administration	PE: AEs and SAEs SE: Pharmacokinetics	– –	–
		E2814	Humanized antibody derived from 7G6 clone; Eisai Co., Ltd	IgG1; Mid-domain aa 299–303; 362–366; MTBR-containing tau protein species	Declined tau-243 fragment; CSF ptau217	NCT04971733; Phase I/ II (Completed); 2021–06 to 2024–05	8 dominantly inherited AD with mild to moderate cognitive impairment	i.v. at set intervals 12 w (phase 1b) + 96 w (phase 2)/ over 52 w	PE: AEs SE: Pharmacokinetics	– –	–
					–	NCT06602258; Phase II (Recruiting, New); 2024–09 to 2027–08	90 early AD patients (Concurrently administered with lecanemab)	E2814 of 4 doses, and placebo + Lecanemab i.v. Q4W (18 m)	PE: CSF MTBR-tau-243 (6 m) SE: CSF MTBR-tau-243; tau-217; [^18^F] GTP1 PET (18 m); ECG; AEs, SAEs, Pharmacokinetics	[^18^F] GTP1 PET imaging/ ECG; CSF MTBR-tau-243; tau-217	–
					–	DIAN-TU; NCT05269394; Phase II/III (Active, not recruiting); 2021–12 to 2028–07	197 early-onset AD patients caused by genetic mutation	Cohort 1: i.v. Lecanemab (0–208 w) + E2814 (24–208 w) Cohort 2: i.v.E2814 (0–208 w) + Lecanemab (52–208 w) placebo: i.v. placebo + Lecanemab/ E2814 (matching period)	PE: tau PET (24, 104, and 208 w) SE: CDR-SB; CSF ptau217/total tau; amyloid PET (24 w); NfL	tau/amyloid PET; CSF ptau217/total tau; NfL	–
		Posdinemab/ JNJ-63733657	Humanized version from JNJ-63733657, PT3; Janssen	IgG1; Mid-domain aa 204–225; Phosphorylated tau pT217	Declined free CSF p-tau 217, p-tau181	NCT04619420; Phase II (Active, not recruiting); 2021–01 to 2032–12	523 AD patients with a positive tau PET scan	Low/ High-dose and placebo i.v. Q4W (104 w)	PE: iADR SE: ADAS-Cog 13; ADCS-ADL MCI; RBANS; CDR-SB; tau PET	[^18^F] GTP1 PET imaging; CSF concentrations of Total, Free, and Bound p217 + tau Fragments	–
		Bepranemab/ UCB0107	Humanized mAb derived from antibody D (234–246); Hoffmann-La Roche, UCB S.A	lgG4; Mid-domain aa 235–250; Monomeric and PHF tau	Show statistically significant effect in SEs (Cognitive: ADAS-Cog 14 & tau PET) Clinical benefit in people with low baseline tau levels and no ApoE4 on all endpoints (CDR-SB, ADAS-Cog14, tau PET, Activities of daily living)	NCT04867616; Phase II (Active, not recruiting); 2021–06 to 2025–07	450 MCI or mild AD (amyloid accumulation by PET or CSF markers)	Low/ High-dose and placebo i.v. Q4W (80 w) + 48 w OLE	PE: CDR-SB SE: tau PET; iADRS; ADASCog14; ADCS-iADL; MMSE	tau PET; –	–
		PRX005/ BMS-986446	– Bristol-Myers Squibb, Prothena	IgG1; Mid-domain in the R1, R2, and R3 repeats; Recognize NFT,regardless of phosphorylation status	–	TargetTau-1;; NCT06268886; Phase II (Recruiting); 2024–03 to 2027–11	475 early AD patients	Low/ High-dose and placebo i.v	PE: CDR-SB (76 w) SE: tau PET; iADRS; ADASCog14; ADCS-iADL; MMSE	tau PET; –	–
		PNT001	Humanized mAb derived from cis mAb clone, 113; Pinteon Therapeutics	lgG4; Cis-pT231; Cis- isomer of pT231 tau	–	NCT04677829; Phase I (Terminated); 2019–09 to 2021–02	TBI patients	Single escalating doses of i.v. administration	PE: AEs; Symptom Other outcomes: CSF total tau, cis pT231 tau, pT231 tau, and NfL, serum NfL	– –	–
	C-terminal	Lu AF87908	Humanized mAb derived from C10.2; Lundbeck	IgG1; pS396; Phosphorylated tau pS396	–	NCT04149860; Phase I (Completed); 2019–09 to 2023–07	86 healthy participants and AD patients	Single i.v	PE: AEs; Pharmacokinetics	– –	–
		RG7345	– Roche	– pS422; Intra-neuronal NFTs	–	NCT02281786; Phase I (Completed); 2015–01 to 2015–10	48 healthy volunteers	Single escalating doses of i.v. administration	–	– –	–
		MK-2214	Immunizing mice with synthetic tau oligomers encapsulated in polymersomes; Merck Sharp & Dohme LLC	– pS431; Pathological tau, including oligomeric and higher molecular weight forms	–	NCT05466422; Phase I (Active not recruting); 2022–09 to 2024–07	48 MCI or mild-to-moderate AD	MK-2214 and placebo i.v. on days 1, 29, and 57	PE: AEs; Pharmacokinetics. phospho-tau in CSF	– CSF phospho-tau	–
		APNmAb005	Immunizing mice with synthetic tau oligomers encapsulated in polymersomes; Aprinoia Therapeutics	– – tau oligomer		NCT05344989; Phase I (Active not recruting); 2020–05 to 2024–07	40 healthy volunteers	Single ascending dose	PE: AEs; Symptom Other outcomes: plasma and CSF tau and ptau-181, 217, and 231	– CSF and Serum ptau-231; ptau-217	–

Data of clinical trials are from ClinicalTrials.gov (https://clinicaltrials.gov)

*PE* primary endpoint, *SE* secondary endpoint, *AEs* adverse events, *SAEs* serious adverse events, *Q2W* every 2 week, *Q4W* every 4 week, *OLE* open labled extention, *MDS-UPDRS* movement disorder society-sponsored revision of the unified Parkinson’s disease rating, *mUMSARS* modified unified multiple system atrophy rating scale score, *PSPRS* progressive supranuclear palsy rating scale, *SE-ADL* Schwab and England activities of daily living score, *CGI-C* clinical global impression of change, *CDR-SB* clinical dementia rating scale—sum of boxes, *MoCA* Montreal cognition assessment, *DaT-SPECT* dopamine transporter imaging with single photon emission computed tomography, *vMRI* brain volumes as determined by magnetic resonance imaging, *PET* positron emission tomography, *ECG* electrocardiogram, *CGI* clinical global impression, *iADRS* integrated Alzheimer’s disease rating scale, *ADAS-Cog13* Alzheimer’s disease assessment scale-cognitive subscale, *ADCS-iADL* Alzheimer’s disease cooperative study-instrumental activities of daily living scale, *ADCS-ADL* Alzheimer’s disease cooperative study-daily living inventory, *MMSE* mini mental status examination, *RBANS* repeatable battery for assessment of neuropsychological status, *NfL* neurofilament light chain

#### Cinpanemab

Cinpanemab, previously known as BIIB054, is a human IgG1 monoclonal antibody and the only N-terminal-targeting antibody advanced to the phase II clinical trial. It specifically binds to residues 1–10 of α-syn and demonstrates an 800-fold higher affinity for aggregated form of α-syn over the monomeric form [126, 127]. Cinpanemab inhibits the propagation of α-syn in a cell-based assay and mitigates the progression of both pathology and motor symptoms in mice following PFF injection [126]. In the phase I trial, this antibody demonstrated good safety and tolerance with selective affinity for aggregated α-syn [127, 128]. The efficacy was subsequently evaluated in the phase II SPARK study (NCT03318523) in a cohort of 357 early-stage PD patients, most of whom had been confirmed with dopaminergic neuron loss by dopamine transporter single-photon emission computed tomography (DAT-SPECT). The study employed a delayed-start design during 72 weeks, in which participants who received placebo in the first year were randomized into one of the active treatment arms in the second year [129, 130]. Despite the robust enrollment, with 96% showing loss of dopamine terminals and 93% with detectable α-syn seeds in CSF samples, cinpanemab did not produce significant improvements in the Movement Disorder Society-Unified Parkinson’s Disease Rating Scale (MDS-UPDRS) total score or its subscores (Parts I, II, and III). Additionally, no significant alleviation was observed in the DAT-SPECT imaging outcomes over 52 weeks compared to placebo. Therefore, cinpanemab failed to reach its primary or secondary endpoints [130, 131]. Furthermore, no significant changes in biomarkers were observed during clear clinical progression, including total α-syn, α-syn seeding, and neurofilament light chain (NfL) levels in CSF and plasma samples [132]. The SPARK study was conducted from January 2018 to April 2021 and terminated due to lack of efficacy.

#### Prasinezumab

Prasinezumab (or PRX002), specifically targeting residues 118–126 of the C-terminus, was the first anti-α-syn therapeutic antibody to enter clinical trials. It demonstrates an over 400-fold greater affinity for oligomeric than monomeric forms of α-syn. Preclinical studies on its murine analog, 9E4, showed reduction of neurotoxic truncated α-syn forms, diminished intracellular α-syn pathology, halting of its propagation, and improvement of motor and cognitive functions in transgenic mouse models of LB diseases [133–135]. Phase I trials confirmed the safety, tolerability, and effective peripheral target engagement of prasinezumab, showing reduction of free serum α-syn by up to 97% at the highest dose of 60 mg/kg in PD patients [2, 136, 137]. In 2017, the phase II PASADENA study (NCT03100149) enrolled 316 newly diagnosed PD patients with mild symptoms. This study was a 52-week placebo-controlled phase followed by another year of blinded antibody treatment [2]. While the primary efficacy endpoint on the MDS-UPDRS was not met, there was a trend towards benefit on the UPDRS Part III that measures motor function decline, and several other pre-specified motor function tests, including mobile-phone-based endpoints [138, 139]. Notably, recent analyses have indicated a 40%–64% reduction in the rate of motor sign progression (UPDRS Part III) in a subgroup of patients with a diffuse malignant phenotype or those using monoamine oxidase B (MAO-B) inhibitors at baseline, suggesting that prasinezumab might be more beneficial for patients with advanced symptoms [3]. As updated at the 2024 AD/PD conference, open-label treatment data from 271 patients after three to four years of treatment indicated that the worsening of the MDS-UPDRS Part II and III remarkably lagged behind that of matched historical controls from the PPMI (Parkinson’s Progression Markers Initiative). The phase IIb PADOVA study (NCT04777331) is underway, targeting PD patients with more advanced symptoms and on stable symptomatic PD medication. This study, which involves monthly prasinezumab treatments, is scheduled to continue for 18 months and conclude in 2024. The recently released data showed its potential clinical efficacy in the primary endpoint, albeit with no statistical significance. The effect of prasinezumab was more pronounced in the population treated with levodopa.

#### Lu AF82422

LU AF82422 is a humanized monoclonal IgG1 antibody that targets the C-terminus of α-syn to prevent the uptake and seeding of aggregates by binding to extracellular α-syn. In phase I trials, LU AF82422 achieved a 37% reduction in free-to-total α-syn ratio in the CSF, indicating effective target engagement [140, 141]. In the phase II AMULET study (NCT05104476) involving 61 patients with MSA, LU AF82422 was administered over 48 to 72 weeks, followed by a 48-week open-label extension period. The most recent results showed a 19% reduction in the rate of decline of the Unified Multiple Systems Atrophy Rating Scale (UMSARS) total score with LU AF82422 treatment. While lacking statistical significance, there were observable trends toward slowing both the primary and the secondary endpoints. Notably, a post-hoc subgroup analysis updated in 2024 at the AD/PD conference revealed a statistically significant 42% slowing of progression in the less impaired subgroup on the modified UMSARS. Additional promising outcomes included reduced brain volume loss in the pons and cerebellum and a significant decrease in the CSF NfL level. Now, the phase III MASCO study of Lu AF82422 in MSA (NCT06706622) is actively recruiting participants, with estimated completion in 2029.

#### MEDI1341

MEDI1341, also known as TAK-341, is a monoclonal antibody targeting the C-terminal epitope 102–130 on both monomeric and aggregated α-syn. It inhibits the cell-to-cell spread of human α-syn fibrils in cell cultures and a mouse model of α-syn pathology propagation. Notably, the efficacy is independent of immune-mediated mechanisms, as TAK-341 and its variant with reduced Fc receptor-binding demonstrated comparable effectiveness [142]. MEDI1341 is undergoing a phase II clinical trial (NCT05526391) involving 138 patients with MSA, scheduled to conclude in August 2025. Patients will receive monthly antibody infusions over one year. The primary endpoints of the trial are motor disability and autonomic function assessed by USMARS Part I.

#### ABBV-0805/Exidavnemab

ABBV-0805 is a humanized monoclonal antibody derived from mAb47 that selectively targets aggregated α-syn by binding to C-terminal residues 121–127. Preclinical studies have shown that mAb47 effectively reduces pathological α-syn levels, alleviates motor dysfunction, and prolongs survival in transgenic mouse models of PD and MSA [143, 144]. Cell-based assays have demonstrated that ABBV-0805 mediates Fcγ-receptor-dependent uptake of soluble aggregated α-syn by microglia and inhibits of neurotoxicity in primary neurons [36]. In addition, passive immunization with mAb47 decreases intracellular α-syn through autophagy-mediated degradation, accompanied by a shift toward the phagocytic phenotype of microglia [144]. mAb47 has also been shown to reduce α-syn aggregates in both prophylactic and therapeutic paradigms in a dose-dependent manner [36]. Moreover, mAb47 reduces pS129 α-syn pathology in the upper brain stem and preserves cognitive functions in both early and long-term treatment models [145]. ABBV-0805 has demonstrated good tolerance and safety in phase I trials and can recognize LBs in post-mortem PD brains. Despite these promising findings, a phase Ib trial involving 32 mild to moderate PD patients was discontinued for strategic reasons (NCT04127695). A new phase II study, EXIST, targeting 24 patients with idiopathic PD, commenced in November 2024. This study aims to assess the safety and tolerability of multiple doses of ABBV-0805 (NCT06671938).

### Current clinical trials of anti-tau immunotherapy

Fourteen anti-tau antibodies have entered clinical trials, with 10 specifically tested in patients with PSP syndrome, AD, and mild cognitive impairment (MCI) [146, 147]. Unlike α-syn mAb, the target epitopes of tau antibodies are across the whole tau protein. The therapeutic strategies can generally be categorized into three mechanistic approaches: (1) targeting all forms of tau to block extracellular tau transmission, exemplified by semorinemab, tilavonemab, and gosuranemab; (2) selective for specific tau conformations to inhibit tau aggregation, such as PRX005 (R1, R2, and R3 repeats within the MTBR), APNmAb005 (targeting synaptic tau oligomers) [148] and tau oligomer-specific monoclonal antibody (TOMA) [149–153]; and (3) specifically targeting phosphorylated tau epitopes to mitigate the pathological effects associated with tau hyperphosphorylation, such as posdinemab (pS217), Lu AF87908 (pS396), PNT001 (pT231), and MK-2214 (pSer413). Below, we primarily focus on the results from completed clinical trials involving the first generation of anti-tau immunotherapies, and provide updates of ongoing trials.

#### Semorinemab

Semorinemab (formerly RO7105705) is the only tau-targeting mAb showing potential positive results in clinical trials. This humanized IgG4 mAb targets tau at amino acids 6–23, binding effectively to both monomeric and oligomeric forms of tau, regardless of the phosphorylation status. This target engagement has been confirmed in both nonhuman primates and AD patients, as evidenced by increased plasma total tau concentrations [154, 155]. In preclinical models, the murine version of semorinemab, muMTAU, significantly reduces tau accumulation in the hTau P301L mouse model, suggesting potential efficacy against tauopathies [154]. In the phase II TAURIEL trial (NCT03289143) involving patients with prodromal to mild AD, semorinemab did not improve clinical outcomes despite reductions of CSF total tau and p-tau181 across all treated groups. Moreover, no impact was observed on the downstream markers of neurodegeneration or inflammation [156]. However, in another LAURIET Phase II study (NCT03828747) targeting patients with moderate AD, semorinemab slowed cognitive decline by 42.2% on one of its two primary endpoints, the Alzheimer’s Disease Assessment Scale-cognitive 11-item (ADAS-Cog11) after 49 weeks. Yet, it did not show improvements on the ADCS-ADL (Alzheimer’s Disease Cooperative Study Group–Activities of Daily Living Inventory) or any secondary endpoints. The treatment with semorinemab did not affect biomarker or tangle accumulation as measured by [^18^F] GTP1 tau PET [157]. Given that the ADAS-Cog improvements were mainly derived from the memory domain, precisely word recognition, cautions remain on the effects of semorinemab on AD pathology [158]. Recent immunoassay studies have identified increased levels of plasma p-tau181 and CSF chitinase-3-like protein 1 in patients following treatment in both phase II studies, along with increased levels of plasma GFAP in the LAURIET study, suggesting that semorinemab may stimulate glial activation in the presence of AD-associated tau pathology [159]. However, other AD pathophysiological biomarkers and complement proteins showed no consistent responses to semorinemab [159, 160].

#### Tilavonemab

Tilavonemab (formerly ABBV-8E12) is a humanized IgG4 antibody that targets the N-terminal amino acids 25–30 of tau protein. It does not need to be taken up into neurons; rather, it enhances the uptake of tau aggregates by BV2 microglia [161]. In cell-based assays, the murine version of tilavonemab, HJ8.5, effectively blocks the seeding and uptake of AD-derived tau in primary neurons and halts the progression of tau pathology [162, 163]. Both systemic and intracerebroventricular administration of HJ8.5 significantly reduce the levels of hyperphosphorylated, aggregated, and insoluble tau in a tau P301S transgenic mouse model. This treatment also decreases tau seeding activity and mitigates cortical and hippocampal atrophy [161, 164]. Additionally, peripheral administration of HJ8.5 decreases brain soluble tau and ISF tau, leading to an increased tau level in the plasma [165]. However, the clinical efficacy of tilavonemab is less promising. A phase II trial ARISE (NCT02985879), which aimed at evaluating tilavonemab in PSP patients, was terminated due to the unsatisfactory results in improving the Progressive Supranuclear Palsy Rating Scale (PSPRS) Total Score, despite the decreased CSF free tau and increased plasma total tau levels [146, 166]. Autopsy from one treated patient showed no pathological changes attributable to the treatment [167]. A further phase II trial (NCT02880956) involving early AD patients with treatment for over 96 weeks plus 16-week follow-up also missed its endpoints. Tilavonemab failed to slow cognitive decline or reduce tau deposition, brain atrophy, or plasma NfL levels [168, 169].

#### Gosuranemab

Gosuranemab (formerly BIIB092, IPN002) is a humanized IgG4 monoclonal antibody that targets the N-terminal amino acids 15–22 of tau. It binds unselectively to monomeric, fibrillar, and insoluble forms of tau. Preclinical testing demonstrated its effectiveness in reducing unbound N-terminal tau fragments in the ISF and CSF of rTg4510 human-tau-transgenic mice and the CSF of patients with PSP and AD. Immunization with gosuranemab reduced seeding-competent AD-tau in immunodepletion assays [170]. In the phase I study, gosuranemab significantly reduced CSF unbound extracellular tau four weeks post-infusion without serious adverse events [171]. It achieved a 90% reduction of free extracellular tau in the CSF of PSP patients, although the total and p-tau levels remained unchanged [172]. A post-mortem study indicated potential glial responses involving tau accumulation within astrocytic lysosomes in frontotemporal lobar degeneration with tauopathy (FTLD-Tau) cases following antibody treatment without clearance of neuropathologic tau inclusions [173]. Despite the promising early results, gosuranemab did not meet the primary endpoint of PSPRS changes in the phase II trial PASSPORT (NCT03068468) in PSP patients, nor did it show benefits in secondary outcomes [174, 175]. Similarly, the phase II TANGO trial (NCT03352557) in patients with MCI or mild AD reported no significant benefits on clinical endpoints, including CDR-SB or tau-PET after 78 weeks of monthly infusions at three different doses. Additional data suggested potential worsening of the ADAS-Cog13 at 18 months [176]. Due to these negative results, another phase 1b placebo-controlled “basket” trial (NCT03658135) targeting four primary tauopathies—CBD, FTLD with *MAPT* mutations, CTE, and non-fluent PPA—was discontinued.

#### Zagotenemab

Zagotenemab (formerly LY3303560) is a humanized IgG4 anti-tau antibody derived from mouse monoclonal antibody MC1. It targets tau residues 7–9 and 312–322, demonstrating a 1000-fold higher affinity for soluble tau aggregates over monomeric tau forms [113]. Peripheral administration of MC1 significantly reduced tau pathology in the JNPL3 mouse model. In the P301S mouse model, MC1 effectively reduced hyperphosphorylated insoluble tau and NFTs levels, while enhancing microglia-mediated tau degradation, thereby significantly delaying the onset of motor function deterioration [112, 177]. No dose-limiting adverse events were observed in the phase Ib study of zagotenemab. While treatment resulted in a dose-dependent increase in plasma tau, tau-PET showed no changes in 22 AD patients [178]. The clinical efficacy of zagotenemab was assessed in a phase II trial PERISCOPE-ALZ (NCT03518073) involving patients with early symptomatic AD for over 100 weeks. Unfortunately, the trial did not meet its primary endpoint, measured by the iADRS (Integrated Alzheimer’s Disease Rating Scale), nor did it show treatment effects in secondary outcomes. No treatment effect was demonstrated by [^18^F] PET, volumetric MRI, or NfL analyses except for a dose-related increase in plasma p-tau181 and total tau, leading to a decision to close the trial [179].

#### E2814

E2814 is a humanized IgG1 monoclonal antibody targeting the MTBR of tau, a key component of tau tangles involved in the seeding and spreading of pathological tau aggregates. E2814 specifically binds to pathological tau structures in the AD brain [6]. Functionally, E2814 binds to the HVPGG motifs in both the R2 and R4 regions of MTBR, inhibiting tau aggregation in vitro and facilitating its clearance by microglia [6]. In preclinical studies, E2814 modestly reduced the propagation of aggregated tau in a K18 P301L mouse model initiated with tau fibril injection, demonstrating its potential to interfere with tau transmission [6]. The binding of E2814 to pathological tau has been confirmed in postmortem brain tissues, including NFTs from AD and PSP patients and Pick bodies from Pick’s disease. It is currently being evaluated in a phase I (NCT04231513) and a phase II (NCT04971733) clinical trial involving patients with mild to moderate cognitive impairment due to dominantly inherited AD, which were completed in 2024. Although detailed results have not yet been published, preliminary reports indicate that the MTBR-tau-243 fragment, an emerging CSF biomarker for tau tangles, declined by 30% to 70% following antibody treatment in AD patients, providing the therapeutic potential of targeting MTBR domains in tauopathies (Clinical Trials on Alzheimer’s Disease conference (CTAD) 2024). Recently, a new phase 2 study (NCT06602258) aimed at concurrent treatment with E2814 and lecanemab was started, recruiting early-stage AD patients. The primary outcome measure for this study was the change of CSF MTBR-tau-243 levels over 6 months. The study is scheduled to conclude in 2027.

#### Posdinemab/JNJ-63733657

Posdinemab, also known as JNJ-63733657, is a humanized IgG1 monoclonal antibody that binds to residues 204–225 within MTBR of tau. It exhibits a high affinity for tau pT217 and phosphorylated PHF. The phase I study reported that posdinemab decreased total p-tau212 and p-tau217 CSF levels in a dose-dependent manner and decreased p-tau181 levels in participants with prodromal or mild AD [180, 181]. Currently, posdinemab is being evaluated in a phase II clinical trial (NCT04619420) targeting patients at early stages of AD to assess its efficacy in slowing or halting the progression of tau-related pathology. This trial is scheduled to be completed in 2025.

#### Bepranemab/UCB0107

Bepranemab (formerly UCB0107) is a humanized IgG4 monoclonal antibody derived from antibody D that targets amino acids 235–250. This antibody recognizes both monomeric and PHF forms of tau [182]. It has shown significant efficacy in blocking tau seeding in cell-based assays, outperforming other tested antibodies, including those targeting the N-terminal (aa 15–24 and 25–30), the C-terminal (aa 312–322), and the phosphorylated sites, such as pS202 + pT205 and pS422 [182]. In tau transgenic mice injected with AD brain extracts and mice injected with K18 P301L tau fibrils, UCB0107 exhibited better performance than antibodies targeting the N-terminus. It effectively neutralized pathological tau forms and blocked the spread of tau pathology [183]. No safety concerns were reported from a phase 1 study involving 25 PSP patients. Currently, a phase II clinical trial (NCT04867616) is underway, aiming at testing further the efficacy of bepranemab in patients with MCI or mild AD. This study is expected to conclude in 2025. Data have been presented recently in CTAD 2024: although it did not show efficacy on primary endpoint CDR-SB in the whole population, bepranemab was the first tau antibody to show statistically significant effect on secondary endpoints (Cognitive: ADAS-Cog 14 & tau PET). Moreover, the antibody had a statistically significant effect on all endpoints (CDR-SB, ADAS-Cog14, tau PET, Activities of daily living) in sub-populations with lower tau pathology at the beginning.

#### Lu AF87908

Lu AF87908, formerly hC10.2, is a humanized IgG1 monoclonal antibody explicitly targeting phosphorylated tau at residue 396 (pS396) [184]. Preclinical studies revealed that the mouse version of Lu AF87908, C10.2, possesses a high affinity for hyperphosphorylated tau aggregates, reducing the seeding capability of brain-derived tau in cultured neurons and rTg4510 tau transgenic mice [184]. Additionally, C10.2 facilitates the uptake and lysosomal degradation of pathological tau aggregates by primary microglia, mediated through the Fcγ receptor of the antibody [185]. The humanized version Lu AF87908 demonstrated similar binding to phosphorylated tau in postmortem brain tissues of AD and other primary tauopathies, effectively preventing further aggregation induced by brain extracts [186]. Intravenous administration of Lu AF87908 leads to a dose-dependent reduction in the CSF level of pS396-tau in rTg4510 mice, providing additional evidence of its potential for clinical use [187]. Currently, Lu AF87908 is being evaluated in a phase I clinical trial in healthy subjects and patients with AD.

#### PNT001

PNT001 is a human IgG4 monoclonal antibody that targets the *cis*-isomer of phosphorylated tau at threonine 231 (*cis*-pT231), a form of pathological tau that increases significantly after traumatic brain injury (TBI) [188] and also identified in brain tissues of patients with AD, vascular dementia, and CTE [189]. This particular *cis*-conformation, modulated by the peptidyl-prolyl *cis*/*trans* isomerase Pin1, promotes tau aggregation due to its resistance to dephosphorylation and degradation [190, 191].

In preclinical models of TBI, the murine version of PNT001, mPNT001, prevented a range of neuropathological outcomes, including axonal pathology, astrogliosis, tau oligomerization, NFT formation, and brain atrophy [192–195]. Furthermore, mPNT001 immunotherapy in a mouse model of VCID (vascular contributions to cognitive impairment and dementia) ameliorated the progression of AD-like neurodegeneration and cognitive impairment, and recovered transcriptomic changes resembling those in elderly patients with AD [196]. Recent research has shown a high binding affinity of PNT001 to NFT-like structures in tauopathy patients. PNT001 reduced tau seeding when incubated with Tg4510 mouse brain homogenates in vitro [195]. It also remarkably alleviated mature NFT formation, improved synaptic and behavioral outcomes, and reduced serum NfL in a mouse model of human tauopathy [197, 198]. A phase I clinical trial demonstrated a linear pharmacokinetic engagement in both blood and CSF, along with good tolerability at all dose levels following intravenous administration of PNT001 [199]. However, another phase I study involving patients with acute TBI was discontinued due to recruitment challenges. Currently, there are no ongoing phase II clinical trials for PNT001.

#### MK2214

MK-2214 is an anti-pSer413 tau antibody targeting pathological tau in AD brains but not healthy ones. In mouse models of tauopathy, MK-2214 effectively reduces levels of hyperphosphorylated tau, tau oligomers, and tangle formation. Additionally, it alleviates synaptic loss and improves memory function [200]. MK-2214 is currently undergoing a phase 1 clinical trial involving a multiple-ascending-dose study in adults diagnosed with MCI or mild-to-moderate AD dementia. The primary focus of the trial is to assess adverse events and pharmacokinetics. This study is anticipated to be completed in May 2025.

### Protein-targeting immunotherapies in co-pathology

A substantial body of evidence suggests that amyloid proteins may contribute synergistically to the etiology of neurodegenerative disorders [71, 201, 202]. Interestingly, evidence from several preclinical studies has shown that antibodies targeting a single protein can significantly affect other pathological proteins within the complex milieu of their co-occurrence [203]. Passive immunization with tau oligomer-specific antibodies TOMAs decreases insoluble and phosphorylated α-syn oligomers in a mouse model of synucleinopathy. The intervention preserved synaptic proteins and dopamine levels in olfactory bulb, and improved both cognitive and motor functions in mice, suggesting that targeting tau may disrupt the detrimental interplay between α-syn and tau [204]. Moreover, TOMAs have previously been demonstrated to mitigate Aβ plaque deposition in Tg2576 AD mice [151]. Similar effects of anti-tau immunization on Aβ have been observed in 3 × Tg-AD mice, in which the N-terminal-targeting tau antibody 43D reduced amyloid precursor protein (APP) levels in the hippocampus (CA1) and amyloid plaques in the subiculum, with a trend of decrease for Aβ40 and Aβ42 levels in the forebrain. Remarkably, this treatment also reduced amyloid plaque loads even in the presence of physiologically endogenous mouse tau with AD-tau seeded pathology [151, 205, 206]. Another study employing combined therapies targeting α-syn and Aβ has demonstrated additive effects in alleviating amyloid pathology and behavioral deficits in APP/α-syn transgenic mouse models. Immunization with anti-Aβ AD02 is more effective in reducing the Aβ burden and p-tau, improving cognitive functions. Meanwhile, PD-AFF1 decreases cholinergic and dopaminergic terminals and motor functional loss, pathologies related to α-syn. Interestingly, AD02 was as effective as PD-AFF1 at reducing α-syn levels, indicating a potential cross-reactive or indirect mechanism [207]. These findings have encouraged the adoption of dual-target strategies or approaches that simultaneously address multiple amyloid proteins [208]. Besides, antibodies targeting the β-sheet-rich structures have effectively improved behavioral outcomes in AD mouse models, with NPT088 currently being evaluated in clinical trials [209–211]. Recent research explored the functional characteristics of small molecules capable of simultaneously targeting monomeric tau and α-syn, or peptides that bind to tau and Aβ [84, 85]. Anle138b, an α-syn oligomer inhibitor, exhibits stable polar interactions within a cavity formed between β-strands and has shown efficacy in reducing tau aggregates and prion protein (PrP) amyloid plaques in mouse models [212–215]. These novel findings underscore the potential of strategies designed to concurrently target multiple amyloid proteins, offering a promising approach for treating neurodegenerative diseases.

## Lessons learned from clinical trials

### Incompatible ammunition-antibody-specific factors

**Fig. 1:**
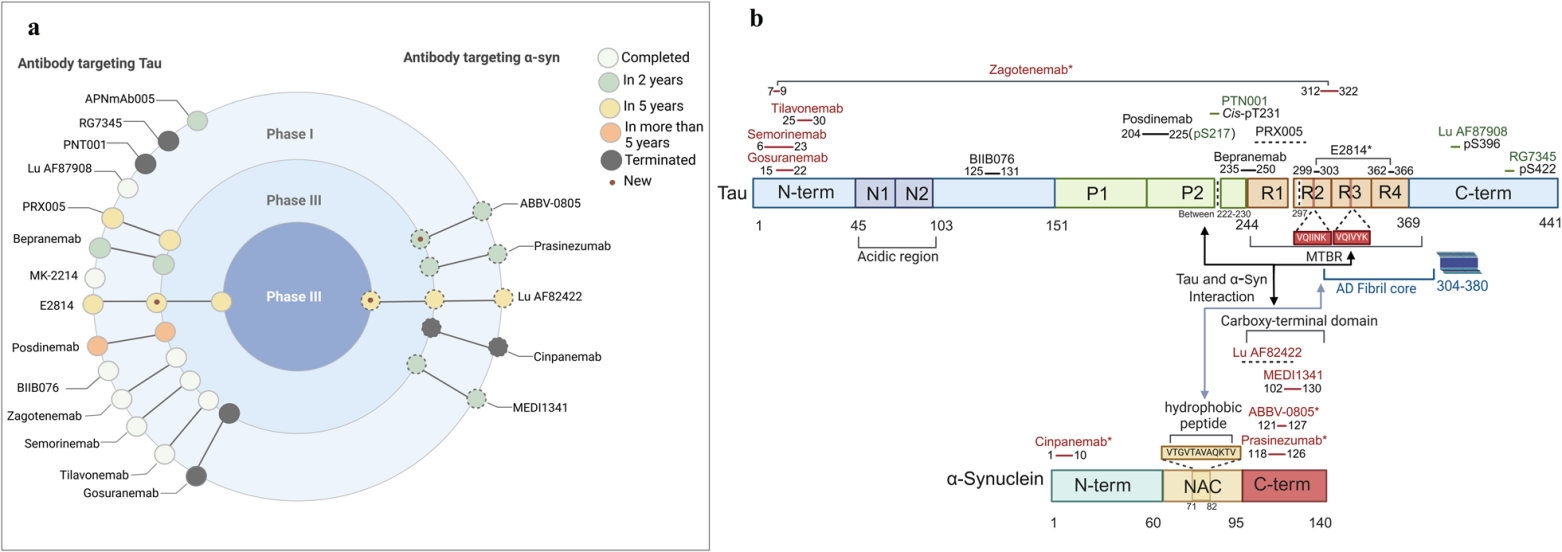
Overview of immunotherapies targeting tau and α-synuclein (α-syn) in clinical development, with their corresponding target epitopes indicated. **a** At the time of writing, five α-syn antibodies are under clinical trials. Fourteen tau antibodies have entered clinical trials, with 10 specifically tested in patients with tauopathies. Among these antibodies, E2814 and Lu AF82422 have progressed to phase III trials. In this diagram, these clinical trials are categorized by their anticipated completion dates. Symbol ‘⊙’ marks clinical trials newly registered on ClinicalTrials.gov. Circles with dashed lines indicate clinical trials that have been terminated. **b** Epitopes of tau and α-syn recognized by the antibodies tested in the clinical trials. Binding epitopes lacking detailed information are represented with dashed lines. In addition, molecular interactions between the two proteins reported in literature are addressed with bidirectional arrows: (1) C-terminus of α-syn and MTBR of tau [76–78]; (2) a specific region in NAC in α-syn (yellow) and hexapeptide motifs (red) of tau [79]; and (3) C-terminus of α-syn and proline-rich region of tau [80, 81]. Cleavage of the tau protein at amino acids 222–230 or around amino acid 279 significantly increases the risk of off-target effects of tau antibodies. The amyloidogenic (AD) fibril core is located around amino acids 304–380 of tau protein

To date, various clinical trials targeting α-syn, tau, and their combined immunotherapies are underway (Fig. 1a). Yet, the majority have not achieved their primary endpoints. Incompatible antibody binding is an important issue that potentially contributes to these failures, despite the confirmation of epitopes and target engagement in most preclinical studies (binding epitope distribution shown in Fig. 1b). The first generation of tau antibodies target epitopes within the N-terminal, far from seeding-inducing MTBR, with truncations occurring between the N-terminal region and the MTBR. Post-mortem examinations following treatment with antibodies like gosuranemab and tilavonemab have shown only minor alterations in tau pathology, raising concerns about the effectiveness of first-batch tau mAbs that recognize linear epitopes at the N-terminal region [173]. Previous studies have found that the tau N-terminal fragments are commonly reduced in post-mortem AD brains due to cleavage by proteases [216]. Moreover, N-terminal-truncated forms of tau, either secreted by induced pluripotent stem cell (iPSC)-derived neurons or present in human CSF, may compromise the clearance of truncated tau seeds by antibodies designed to target this region [64, 217, 218]. Recently, a cleavage-specific monoclonal antibody 12A12, which selectively binds to N-truncated tau (a neurotoxic 20–22 kDa NH_2_-derived peptide), has been found to significantly improve AD-like behavioral and neuropathological syndromes in Tg2576 animals, providing a promising immunotherapy strategy for AD [219–221].

Binding challenges also arise for α-syn immunization due to modifications in C-terminus such as pS129 and pT125, or truncation at residues 133 or 135, which lead to off-target effects and strain-specific proteolytic patterns [222, 223]. A recent flow cytometry study has highlighted that antibodies recognizing the NAC region of α-syn offer higher specificity and sensitivity than those targeting the C-terminal [224]. Yet, no α-syn mAb targeting the NAC region has reached clinical trials. In contrast, the second batch of tau mAbs focuses more on the mid-region and MTBR. Preclinical data suggest that these antibodies more effectively interfere with the propagation of pathogenic, aggregated tau than those targeting the N-terminal region [225]. This strategy is expected to be promising, given the observation of higher abundance of MTBR-containing peptides in insoluble protein fractions in AD cohorts.

Despite missing of primary clinical endpoints, immunotherapies targeting specific tau epitopes have demonstrated reductions in certain imaging and biofluid biomarkers. For instance, bepranemab significantly reduces pathology as evidenced by tau PET. Immunization with E2814 antibody, which targets epitopes spanning the amyloidogenic fibril core (Fig. 1b), decreases the level of the tau-243 fragment in the CSF. Similarly, posdinemab targeting the mid-region decreases levels of CSF p-tau217 and p-tau181 in participants. A previous ASO therapy targeting the *MAPT* gene (BIIB080) demonstrated that reductions in CSF p-tau181 levels correlated with a slowing of cognitive decline [226]. Thus, alterations in these biomarkers may imply a potential therapeutic benefit from these antibodies. Given the critical role of MTBR in α-syn and tau interaction, blocking this domain not only inhibits self-aggregation of tau but may also mitigate cross-seeding between the two proteins. Besides, exploration of antibodies targeting specific phospho-tau epitopes, such as pS396 and pS422, is also promising. Nevertherless, these antibodies may have limited potential to capture the non-phosphorylated MTBR aggregates, which are still capable of initiating seeding processes.

The failure of antibody targeting of intracellular proteins and their backbone modification in clinical trials may be related to other factors. Several preclinical studies targeting α-syn and tau have observed antibody/protein complexes inside neurons or microglia [227, 228]. The α-syn oligomer/protofibril-selective mAb47 antibody is internalized via the Fcγ receptor [229], while the complex formed by tau seeds and an antibody targeting p-tau422 is taken up through the Trim21 receptor and subsequently degraded [230, 231]. Nevertheless, it remains unclear how effectively these antibodies can reach and impact critical target sites in the brain parenchyma, such as neuron terminals. A recent study using a QSP (quantitative systems pharmacology) model indicates that while substantial target engagement occurs in the CSF, it significantly diminishes in the synaptic cleft, with presence of only 1% to 3% of tau and α-syn antibodies [232]. Furthermore, studies of tau kinetics suggest that the dwelling time of extracellular tau in the synaptic cleft may be too shortfor antibodies to neutralize most tau seeds effectively [218]. Both findings indicate that the low efficacy of these antibodies substantially limits their effectiveness in immunotherapy.

Additionally, the diminished effector function may contribute to the failure of the first batch of four tau antibodies, which are primarily of the IgG4 isotype. IgG4 is known for its lower affinity for Fcγ receptors and is specifically designed to avoid activation of microglia [233]. Fixemer et al*.* have highlighted strong associations between microglial alterations and the subfield high burdens of phosphorylated tau and α-syn, underscoring the crucial role of microglia in modulating immunization against both proteins [234]. This design may inadvertently compromise the glial response necessary for effective macrophage-mediated clearance of protein-antibody complexes, potentially impacting the removal of pathological proteins.

### Translation of pre-clinical findings into clinical trials

Protein-targeting immunization strategies have shown promising efficacy in preclinical tests (Table 2); however, their success has yet to be consistently replicated in clinical trials. This highlights the significant challenges in translating immunotherapies from laboratory models to humans. Various factors may contribute to the discrepancies in trial outcomes, including differences in animal models, cohort selection, dosage regimens, and methods for efficacy evaluation.

**Table 2:** Preclinical studies of antibodies targeting α-syn and Tau on rodent models

	Antibody in clinical trial	Antibody/Clone	Target	Injection, frequency, duration, dose	Animal model	α-Syn pathological effects	Neuronal effects	Other non-neuronal effects	Behavioral effects	Refs.
Anti-αsyn mAb	Cinpanemab/BIIB054	12F4 (IgG2a)	N-terminal (1 − 10)	3 doses prior to PFF inj. + i.p., weekly for 3 m (30 mg/kg)	PD: Wild-type C57BL/6 mice; α-syn A53 T (M83); BAC α-syn A53 T	↓ Truncated/full length α-syn;↓ pSer129 α-syn levels;	↓ DAT loss in striatum;	_	Delay paralysis; ↑ Wire hang performance	[126]
	Prasinezumab	9E4 (IgG1)	C-term. 118–126	i.p., weekly for 6 m (10 mg/kg)	PD/DLB: PDGFb α-syn mice (line D)	↓ FL α-syn in neocortex neuropils; Reduced corpus callosum-α-syn in neocortex (intra-neuronal and neuropil), Hippocampus (intra-neuronal/neuropil); ↓ Reduced insoluble α-syn oligo.; ↓ soluble α-syn mono/oligo.; ↓ insoluble α-syn mono/oligo	↑Synaptic densities; ↑pre-synaptic terminals; ↑PSD95; ↑Synapsin	↓Astrogliosis in PFC	↓Rotarod time; ↓Path in Morris water maze	[135]
		5C1 (IgG1) (9E4 analog)		i.p., weekly for 6 m (10 mg/kg)	PD: Thy1-α-syn mice (line 61)	↓ α-Syn and α-syn aggre. (temporal and striatal neuropil); ↓ axonal α-syn (striatum)	↓ TH loss in striatum; ↑ synaptophysin + MAP2 (neocortex and striatum)	↓Astrogliosis ↓Microgliosis	↓Memory and learning deficits; ↓Error on transversal beam	[133]
		9E4 (IgG1)								
	MEDI1341	MEDI1341 (IgG1)	C-term	Post 1 w PFF inj. i.p., weekly for 13 w (20 mg/kg)	PD: Wild-type C57BL/6 mice + intrahippocampal injection of LV-αsyn	↓ Contralateral and ipsilateral αsyn (hippocampus); ↓ contralateral axonal αsyn (neocortex) ↓ interstitial fluid αsyn levels ↓ CSF fluid αsyn levels ↓ αsyn positive neurons (neocortex and hippocampus)	_	_	_	[142]
	ABBV-0805	mAb47 (IgG1)	Oligo, C-term. 121–127	i.p., bi-weekly for 3 m (20 mg/kg)	MSA: PLP αSyn transgenic mice	↓ Soluble and insoluble α-syn (hippocampus); ↑ α-syn in spinal cord; ↑ pS129 α-syn (SNpc, pontine nuclei and inferior olives); ↑ Co-localization of LCS (autophagy) and pS129 α-syn	↑ Microgliosis; ↑ activated MG; ↑ Iba-1 and olig-α-syn co-localization	_	_	[144]
		mAb47 (IgG1)	Proto fibril	i.p., weekly for 3.5 m(10 mg/kg)	PD: Thy-1 h[A30P] α-syn mice	↓ α-Syn protofibrils in spinal cord	_	_	_	[143]
		ABBV-0805/mAB47 for murine experiments	Humanized mAB47, binding to C-term. 121–127	i.p., weekly starting at age 12 m, (10 mg/kg)	PD: Thy-1- h[A30P] α-syn tg mice	_	_	_	↑ Mean survival from 84 to 160 days	[36]
				i.p., weekly starting at age 12 m, until severe motor deficits (20 mg/kg)	PD: Thy-1- h[A30P] α-syn tg + i.m PFF inj. gastrocnemius after mab treatment	_	_	_	↑ Mean survival from 84 to 95 days	
				Starting 4 w prior to PFF inj.; weekly mab inj. Prophylactic: 2–4 m, until severe motor deficits (20 mg/kg)	PD: Thy-1- h[A30P] α-syn tg + gastrocnemius PFF inj. 1 μg i.m	↓ Soluble and insoluble α-syn (brain); ↓ insoluble pS129-α-syn; ↓ CSF pS129-α-syn; ↓ LB-509 α-syn inclusions (reticular nucleus); ↑pS129-α-syn inclusions (midbrain)	_	_	_	
				Post 2 w after PFF inj.; weekly mab inj. Therapeutic: 2–4 m, until severe motor deficits (20 mg/kg)		↓ Soluble and insoluble α-syn (brain); ↓insoluble pS129-α-syn; ↓CSF pS129-α-syn; Dose-dependent soluble and insoluble α-syn (brain); ↓soluble α-syn at low mab administration (0.25 mg/kg); ↓LB-509 α-syn inclusions (reticular nucleus); ↑pS129-α-syn inclusions (midbrain)	_	_	_	
				Weekly 4 m (20 mg/kg) for 15 m	PD: A53T +/-mice (M83); α-syn tg olfactory bulb injections	↓pS129- α-syn pathology spreading to the contralateral hippocampus (CA1) (58%)	_	_	_	
				i.p. weekly starting at age 6w (20 mg/kg), for 10 m	PD: (Thy-1)-h[A30P]	↓ pS129- α-syn pathology in the upper brain stem	_	_	↓ Recognition and risk assessment deficits at 6 m	[145]
	Semorinemab	muMTAU muMTAU-DANG(effectorless version IgG2a)	N-term 6–23	Start at 13 w i.p. weekly for 13 weeks (3,10,30 mg/kg)	Thy1-hTau.P301L	↓ Tau accumulation and phorylated tau (pTau212/214; pTau202/205)	↓Uptake of oligomeric tau by neurons and shows neuroprotective activity	No hippocampal gliosis observed	-	[154]
	Tilavonemab	HJ8.5	N-term 25–30	Start at 6 m i.c.v. continuously infused for 3 m (7.2ug/day)	Tauopathies: Prnp-MAPT*P301S mice (PS19)	↓ Phosphorylated tau (piriform, entorhinal cortex and amygdala) ↓ Detergent-insoluble tau; ↓Seeding Activity	↓ Phosphorylated Tau in neuropil	↓ Microgliosis	↓ Contextual Fear Deficits	[164]
				Start at 6 m i.p., weekly 3 m (50 mg/kg)		↓ Phosphorylated and thio-S-positive tau (hippocampal CA1 cellular layer); ↓ Detergent-insoluble tau;	↓ Loss of cortical and hippocampal tissue volumes	↑ Uptake of P301S tau aggregates in In BV2-microglial cells (in vitro)	↓ Motor/sensorimotor deficit (Ledge and inverted screen tests; Contextual Fear Deficits)	[161]
				Start at 3, 6, 9 m i.p. (50 mg/kg)	Tauopathies: Prnp-MAPT*P301S mice (PS19); (AAV-syn-P301S),	↓ Brain soluble tau and interstitial fluid tau; ↑ Plasma tau (reflecting soluble, extracellular tau in the brain) Less increase in plasma tau after treatment in 9-month- mice	_	_	_	[165]
Anti-Tau mAb	Zagotenemab	MC-1; PHF-1	7–9 and 312–322	JNPL3: i.p. 3 times a week for 2 m (15 mg/kg) + twice a week for 2 m (10 mg/kg) P301S: i.p. twice a week (15 mg/kg)	JNPL3 (P301L) and P301S mice	↓Hyperphosphorylated insoluble-tau NFT (cortex, spinal cord and brain stem) Numbers of neurospheroids in the spinal cords (P301S mice)	↓Neurofilament-positive neurospheroids in the spinal cord (P301S mice)	No glia activation	↓ Body weight loss and its onset ↑ Fall latency on Rota-Rod	[112]
	E2814	7G6	MTBR 299–303; 362–366	i.p. single immunization piror and post seeds administration (40 mg/kg)	P301S transgenic mice + Intrahippocampal injected human 2 N4R P301S tau seeds	↓Deposition of sarkosyl-insoluble tau (NFTs);	_	_	_	[6]
	Lu AF87908	hC10.2	pS396	A single i.v. of the hC10.2 (1, 60, or 120 mg/kg)	rTg4510 transgenic mice	36% and 48% CSF pS396-tau reduction at 60 and 120 mg/kg	_	_	_	[187]
	PNT001	Murine PNT001 (mPNT001)	*cis* pT231	Weekly i.p., for 1.5 m (20, 60 mg/kg)	rTg4510 transgenic mice	↓Tau12 and total insoluble pT231 tau (AT180) (60 mg/kg); ↓ThioS staining and reduced the size and number of NFTs; ↓ Total insoluble tau	↑long term-potentiation; ↓ serum NfL (20, 60 mg/kg)	↓Neuroinflammatory mRNA transcripts	↑Acquisition and retention (60 mg/kg)	[197]
	APNmAb005	APNmAb005	Oligomeric Tau	Twice daily i.p. for 12 w (10,50 mg/kg)	rTg4510 transgenic mice	↑pTau (AT8), synaptophysin, and extracellular soluble tau (50 mg/kg)	↓Neuronal loss in hippocampus; ↑Tau-expressing neurons	_	_	[148]
	MK-2214	TA1505 antibody (effectorless version IgG2a)	pS413	Weekly i.p. for 5 w (0.1, 1 mg)	Tau 609 and Tau784 mice	↓Tau hyperphosphorylation, tangle formation,	↓Neuronal losssynapse loss	_	↑Spatial learning and memory in Morris Water Maze test	[200]
	_	12A12	N-ter 26–36	Two weekly i.v. Start at 3 m; 6 m (30 ug)	Tg2576; 3xTg mice	↓Pathological tau and APP/Aβ processing metabolisms	↑Synaptic plasticity; ↓loss of dendritic spine connectivity in pyramidal neurons; ↓electrophysiological deficits in hippocampal long-term potentiation; ↓mitochondrial alterations	↓Reactive gliosis	↑Novel object recognition and object place recognition	[219]
	_	TOMAs	Selectively binds oligomer tau	Single dose 30 μg i.v.; i.c.v.at 8 m	P301L (JNPL3)	↓Tau oligomers; phosphorylated NFTs or monomeric tau;	_	_	↓Locomotor and memory deficits	[150]
				Single 60 μg	Brain-derived tau oligomers intracerebrally in 3-month-old wild-type and Htau mice	↓Accumulation of tau oligomers	_	_	↓Cognitive deficits for up to 1 month	[149]
				Biweekly injections of 60 μg		Oligomeric tau	_	_	↑Memory function	
				Biweekly with 60 μg TOMA i.p. from 3.5 to 8 months of age	Tg4510 (cognitive difects 3.5 m)	↑Oligomeric tau was in CSF and plasma; ↑mouse IgG	_	_	No change behavioral deficits	[90]
				Single i.v. of 120 μg/animal	Aged Htau and JNPL3	↓Tau oligomers	_	_	↑Cognitive phenotypes	[153]
				Single i.v. of 30 μg/animal	Tg2576 mice	↓Tau oligomers; ↓Aβ oligomers (A11); ↓Aβ 56; ↑Aβ (OC); ↑amyloid plaques in cortex and hippocampus	↑Mushroom spines; ↓GSK3 activity	_	Improved memory; no difference in freezing behavior	[151]

Commonly used mouse models for testing α-syn and tau therapies fall into two main categories: (1) genetically modified rodent models that overexpress mutated human α-syn or tau, such as A53T (M83), A30P under different promoters (PLP/Thy-1) [133, 134, 143, 144], and tau P301L/S, which predominantly models for FTLD rather than AD [165, 235]; and (2) rodent models inoculated with patient-derived or in vitro*-*produced PFFs to induce endogenous α-syn and tau aggregation, mimicking the protein aggregation and propagation processes observed in different neurodegenerative diseases [126]. Nevertheless, these short-term models may fail to accurately replicate the complex pathology arising from long-term protein accumulation and interactions among multiple amyloid proteins, particularly in sporadic cases of synucleinopathies and tauopathies that lack clear genetic links to dominant mutations in α-syn or tau [35, 111, 235]. Furthermore, protein aggregation induced in these models often fails to represent the pathological features observed in patients with different types of synucleinopathies and tauopathies [111, 236]. For instance, intracerebral injections of MSA brain extract into transgenic mice expressing human α-syn mutant results in the transmission of pathology. Conversely, administering PD brain extracts to similar models does not induce LB pathology in the recipient animals [110, 237]. Furthermore, in the PLP-promoter α-syn overexpression model, pS129 α-syn in oligodendrocytes is detected in the soluble monomer/oligomer form rather than as fibrillar aggregates, unlike that in MSA or PD patients [111]. In P301L/S tauopathy models, tau aggregation is driven by progressive phosphorylation within the proline-rich domain and the C-terminus, while ubiquitination and acetylation, characteristics of early-stage AD in humans, are not represented in this mouse model [235].

Challenges of tauopathy modeling also include the inability to replicate human 3R or 3R/4R mixed tauopathies, as adult mice only express 4R tau isoforms [238, 239]. Such differences make it difficult to translate findings directly to human conditions. Additionally, the dosage regimen and timing of interventions in these models often do not align with clinical settings. Typically, antibodies are administered in rodent models immediately after symptom onset (around 6–8 months) and continued for 3–6 months [134, 135, 143, 154, 164]. This duration corresponds to a significant portion of a rodent’s lifespan, in stark contrast to one or two-year clinical treatments for patients, even in the prodromal or early stages of synucleinopathies and tauopathies [138, 157, 168, 174]. Preclinical data from Yanamandra et al*.* revealed that mitigation of tau pathology by HJ8.5 (tilavonemab) in 9-month-old PS19 mice was significantly less effective compared to younger mice (3- and 6-month-old), underscoring the importance of early intervention [165]. This hypothesis is supported by a recent *post-hoc* subgroup analysis from the Lu AF82422 treatment reported in the 2024 AD/PD conference, where a statistically significant slowing of progression was observed in the less impaired MSA subjects. However, clinical expectations might not be as optimistic as those observed in animal models, especially regarding the prophylactic effects noted in models induced by PFF administration. In these models, antibody treatment is initiated before the inoculation of PFFs, potentially targeting artificially introduced species rather than the endogenously generated aggregates involved in actual disease progression [36, 240, 241]. These preclinical challenges underscore the difficulties inherent in demonstrating a clinical effect of disease-modifying approaches and highlight the critical need for alignment between preclinical models and clinical trial designs.

Currently, PD and AD patients comprise the largest cohorts for testing therapeutics targeting α-syn and tau [240–242]. Overall, there are no significant concerns regarding the selection of the study population in current clinical trials, as α-syn SAA and tau-PET imaging reliably serve as essential standards for patient inclusion [37, 243, 244]. However, the heterogeneity of participants potentially affects treatment efficacy. A pooled analysis of trials involving amyloid-targeting antibodies, such as lecanemab and aducanemab, showed greater efficacy in improving CDR-SB scores among *APOE* ε4 carriers compared to non-carriers [245]. A recent in vitro study indicated that delivery of tau antibodies across the BBB is particularly compromised in *APOE4* carriers, highlighting the impact of genetic background on drug susceptibility [246]. Additionally, Hamlin et al*.* identified tau antibody labeling signatures associated with Braak stages among different NFT populations in AD patients. The immunophenotype of these tangles shifts from “AT8 high” or “4R tau high” to “3R tau-MN423 high” as they mature, indicating variable binding efficiency among antibodies related to heterogeneity of tau tangles [247]. The risk of poor targeting is compounded by the complex conformational and structural heterogeneity observed among aggregate strains isolated from brain tissues with different synucleinopathies and tauopathies [22, 69, 91, 248]. A latest clinical study found a lower binding affinity of α-syn and tau antibodies to the α-syn/tau co-pathology in AD-LB cases compared to pure AD and PDD. This further indicates that co-pathology can significantly affect antibody binding efficiency and therapeutic outcomes [247]. These findings imply the need to develop therapeutic antibodies that are finely tuned to target specific pathological conformations and stages, enhancing both their efficacy and specificity.

Cognitive and motor functional endpoints are commonly employed in clinical trials to assess symptomatic efficacy of treatments targeting α-syn and tau. Despite the significant alleviation of pathological behaviors observed in mouse models, no completed trials have fully met these primary and secondary endpoints, even though the involvement of these antibodies in targeting plasma and CSF α-syn and tau is well-documented [129, 138, 157, 168, 176, 179]. Interestingly, recent *post-hoc* analyses from the PASADENA study of prasinezumab revealed significant reductions in MDS-UPDRS III scores in prespecified PD subgroups characterized by faster motor progression and on stable use of MAO-B inhibitors at baseline. These findings suggest that the current rating scales may be sensitive or appropriate for patients with more advanced symptoms [3]. Similar trends were noted in the LAURIET trial of semorinemab, which showed slowing of cognitive decline as assessed by the ADAS-Cog11 in patients with moderate AD but not in those with prodromal to mild symptoms [157, 158].

The sensitivity of existing diagnostic standards has long been debated, particularly regarding their ability to evaluate changes at earlier disease stages accurately. For instance, seven of the 11 ADAS-Cog-11 tasks show severe ceiling effects in discriminating between MCI and healthy samples [249–252]. A meta-analysis has indicated that the ADAS-Cog does not significantly differentiate the efficacy of anti-Aβ therapies. This suggests that these scales may not fully capture the nuances of cognitive function in immunotherapy contexts [253]. Likewise, a psychometric analysis based on Rasch Measurement Theory identified a floor effect in the MDS-UPDRS-II and III for early-stage PD patients. This indicates that these measures may lack the precision to accurately measure motor symptoms and their impact on daily activities [254]. MDS-UPDRS III lacks clinometric soundness when combined with other parts [255], raising potential concerns about the use of MDS-UPDRS I + III scores as the endpoints in some clinical trials. Additionally, clinical endpoints involving scales and questionnaires are subject to inter-rater variability. These assessments can be confounded by the effects of symptomatic medications, such as the involuntary dyskinesias induced by Levodopa, dopamine agonists, MAO-B inhibitors, and COMT (catechol-o-methyltransferase) inhibitors [256–258]. These factors may lead to inappropriate interpretations of trial outcomes, potentially hindering successful clinical translation, especially when positive results lack statistical significance.

## How can we make the diseased protein-targeting work?

Firstly, it is crucial to employ refined preclinical models that closely mimic human pathology. Using cellular and animal models with patient-derived α-syn or tau proteins can significantly improve the accuracy of preclinical testing of immunotherapies [259, 260]. Techniques like direct trans-differentiation of skin fibroblasts into neurons preserve the aging transcriptional signature, providing a more accurate representation of human aging and neurodegenerative progression [261, 262]. Additionally, 3D organoids derived from patient-specific iPSCs can recapitulate certain features of α-syn and tau pathology, offering more profound insights into the cellular and molecular dynamics [263–265]. For tau immunization, genome-edited mouse models that express both 3R and 4R tau isoforms can better replicate associated pathologies [238, 239, 266]. Moreover, double transgenic mice, such as the novel humanized Tau‐FAD mice (5 × FAD × hTau-KI) and crossbreeds of *MAPT* knock-in with *APP* knock-in mice, can more accurately mimic the complex interactions between Aβ and tau pathology, allowing for more realistic assessments of anti-tau therapies [267, 268]. In addition, employing direct administration approaches, such as intracerebroventricular or intra-hippocampal injections, will overcome limitations from the low BBB permeability [269, 270]. Optimization of antibody doses, intervention durations, and treatment timings in non-human primate models could significantly bridge the translation gap, enhancing the potential of clinical success in treating neurodegenerative diseases.

Patient screening, selection, and stratification are essential for successful clinical trials. Transplantation of dopaminergic neurons leads to significant improvements in motor tests in PD patients under 60 years of age, underscoring the influence of age on the efficacy of the intervention [271, 272]. Patient stratification based on covariates such as sex, genotype variants (particularly *APOE* status), disease staging, and baseline clinical severity, is crucial for enhancing the accuracy of analysis results [241, 242, 245, 273]. Detailed subgroup analyses can reveal differential therapeutic responses across populations, providing essential insights into the biological implications of trial results [3, 274]. Given these considerations, targeting young early-onset or presymptomatic individuals, especially those carrying *SNCA* or *MAPT* mutations, can clarify disease etiology and assess interventional impacts on disease progression. This approach also supports longer interventions and follow-up periods, as evidenced by the promising results in the open-label extension of the PASADENA trials. Remarkably, biomarker strategies primarily benefit the inclusion of presymptomatic individuals and further stratification of the participants to identify subjects most likely to benefit from the treatment [5, 42, 275, 276]. Both CSF and plasma p-tau markers (e.g., p-tau181, p-tau217, and p-tau231) show great value in the diagnosis of prodromal AD and differentiating AD from non-AD primary tauopathies (e.g., PSP or CBD), as well as other non-AD neurodegenerative disorders (e.g., DLB or behavioral variant frontotemporal dementia) [5, 58, 277, 278]. Recent applications of unbiased machine-learning algorithms have identified diverse PET-based tau spreading subtypes in the typical, memory-predominant clinical spectrum of AD [4]. As the most widely used biochemical test for objective diagnosis of synucleinopathies, α-syn-SAA has successfully detected α-syn aggregates in the CSF of prodromal cases of PD or DLB, including idiopathic rapid eye movement sleep behavior disorder (iRBD), MCI, and pure autonomic failure [42, 279–281]. In another study using CSF samples from the AD Neuroimaging Initiative (ADNI), α-syn-SAA demonstrated its potential in detecting LB co-pathology. Individuals testing positive for SAA experienced more rapid cognitive decline and earlier symptom onset, providing further evidence that co-pathology may significantly influence the efficacy of immunotherapy [282]. This aligns with clinical trials of donanemab, an Aβ-targeting therapy, which displayed better outcomes in patients with lower to medium tau co-pathology compared to those with high tau levels [283]. Therefore, concurrent assessment using multiple biomarker approaches— including α-syn-SAA and ultrasensitive tau-SAA [284], along with tau-PET and amyloid-PET scans—offers a detailed stratification based on pathological amyloid burdens, potentially enhancing the precision of patient selection and improving therapeutic outcomes.

Lastly, unlike CSF and blood biomarkers that benefit monitoring of early-stage disease progression, PET imaging can help monitor the pharmacodynamic effects of treatments by showing aggregate removal or prevention of pathological spread. Second-generation tau PET tracers, such as [^18^F]MK6240, [^18^F]RO948, and [^18^F]PI2620, allow for detection and quantification of longitudinal changes of tau aggregation with higher affinity and selectivity [5, 276, 285, 286]. Moreover, recent advancements have introduced promising PET tracers that selectively bind to α-syn aggregates. For example, [^18^F] ACI-12589 has demonstrated high specificity for α-syn in MSA patients, distinguishing them from other neurodegenerative disorders [287]. Another tracer, [^18^F]-F0502B, exhibits a strong binding affinity for α-syn aggregates with minimal interaction with Aβ and tau, highlighting its potential for imaging synucleinopathies [288]. Additionally, longitudinal studies on patients with dominantly inherited AD have recently revealed distinct evolving signatures of well-utilized biomarkers across different stages of disease progression [289, 290]. Interestingly, despite the lack of symptom alleviation in trials of semorinemab and gosuranemab, a reduction in CSF p-tau217 (by approximately 27%) was observed, mirroring results from amyloid-targeting trials where clinical deterioration was mitigated [5, 157, 176]. These results highlight the need for more comprehensive biomarker panels to evaluate treatment efficacy at various disease stages and contextualize their utility in each therapeutic modality. Additionally, identifying alternative biomarkers beyond p-tau is crucial for better assessing novel-batch mAbs targeting MTBR while avoiding specific epitope engagement. Downstream biomarkers, such as NfL and GFAP, and tracers of dopaminergic neurons and glia, serve as valuable tools for directly reflecting functional improvements [291].

## Conclusions and future perspective

**Fig. 2:**
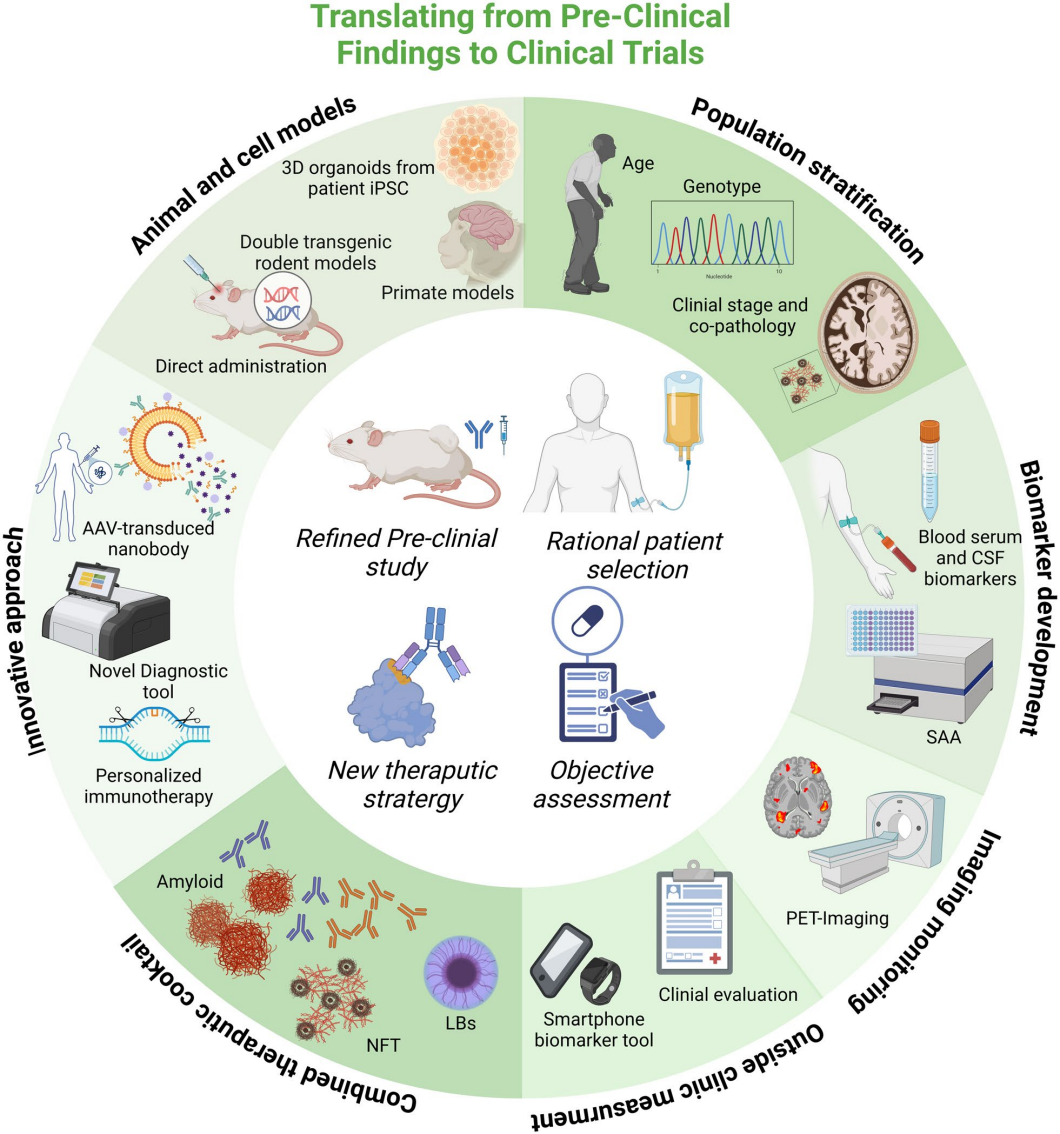
Strategies for successful translation of preclinical findings to clinical trials. This figure outlines four essential strategies to enhance the translation of pre-clinical findings into successful clinical trials targeting tau and α-synuclein, including the use of refined animal and cellular models in preclinical testing to more accurately mirror human neurodegenerative diseases; advanced drug delivery systems to improve antibody efficacy; detailed patient stratification and biomarker development (both body fluid and imaging biomarkers) in clinical trials, which will aid in more precise participant inclusion and objective treatment assessment; and innovative clinical evaluation tools such as smartphone apps for continuous patient monitoring. Innovative approaches—ranging from novel diagnostic assays to personalized immunotherapies will benefit future studies. Particularly, combinational therapies targeting multiple pathologies, such as Lewy bodies (LBs), neurofibrillary tangles (NFTs), and amyloid plaques, represent promising directions for future passive immunotherapy research in synucleinopathies and tauopathies

Preclinical studies have highlighted the potential of immunotherapies targeting α-syn and tau proteins in modulating their pathological aggregation, thereby delaying the progression of synucleinopathies and tauopathies. However, translating these results into successful clinical application is challenging, with minimal improvements observed in clinical trials to date. Four key considerations play critical roles in the translation, including the selection of animal models, therapeutic strategy, cohort selection, and clinical assessment (Fig. 2). Several innovative approaches are emerging as promising tools for targeting intracellular α-syn and tau. In preclinical testings, single-chain antibodies (scFvs) and single-domain antibodies (nanobodies) have demonstrated enhanced BBB permeability and ability to target cryptic epitopes that are inaccessible to whole antibodies [292, 293]. Other engineering improvements, such as modifications with cell-penetrating peptides, are being explored to facilitate the translocation of these antibody fragments across cell membranes, enhancing the clearance of intracellular α-syn and tau pathologies [294, 295]. Gene therapy approaches, such as adeno-associated virus (AAV)-mediated antibody expression, enable sustained intracellular expression of tau-targeting scFvs (as intrabodies), ensuring continuous therapeutic effects [296–299]. These novel approaches for intracellular targeting significantly enhance the efficacy of current passive immunotherapies, enabling more therapeutic interventions for misfolded or aggregated proteins in neurodegenerative diseases. Additionally, head-to-head comparisons between biofluid and neuroimaging biomarkers are needed for biomarker development to establish the optimal prognostic performance across diverse patient populations. Advanced diagnostic tools, including bioassays and imaging techniques capable of identifying pathological conformers, are essential for evaluating therapeutic efficacy more accurately. Emerging technologies, such as the soluble oligomer binding assay, show promise in distinguishing AD from other dementias by detecting Aβ oligomers, and may also be adapted for the detection of α-syn oligomers in PD and DLB [300]. Besides, integrating genomic, proteomic, and transcriptomic analyses could advance our understanding of the biological pathways that regulate protein aggregation and propagation, facilitating the development of personalized immunotherapeutic strategies [276]. Finally, some new therapeutic strategies are focusing on targeting different toxic species of pathological proteins or simultaneously targeting multiple amyloid proteins—representing a future direction for passive immunotherapy. A recent study characterized the pathological diversity in LB diseases using an expanded antibody panel that targets various sequences and PTMs of α-syn, highlighting the promise of combining antibodies that target different epitopes [23]. Additionally, the development of single multivalent and biparatopic antibodies could provide a viable solution for targeting different polymorphs or strains of the same protein. For example, MT3.1, a newly developed multivalent tau nanobody, binds to multiple forms of tau and demonstrates increased affinity for pathological fibrils [301]. Similarly, a single antibody designed to target shared amyloid structures between oligomers and aggregates has shown effectiveness [209, 302]. Given the pathological continuum observed in amyloid-related neurodegenerative diseases, including synucleinopathies and tauopathies, combined therapies that target multiple amyloid proteins simultaneously may offer more effective treatment options. A recent phase 2 study starting in September 2024, explores the concurrent use of the tau-targeting antibody E2814 and the amyloid-targeting antibody lecanemab in early-stage AD participants (NCT06602258). Likewise, cocktail therapies and novel bispecific antibodies targeting both α-syn and tau are anticipated to be evaluated in future trials [203, 208]. Identifying and targeting specific interactive epitopes between these proteins could lead to the development of bi-antigenic agents that bind simultaneously to both proteins. Such an approach holds promises for inhibiting their cross-seeding and improving therapeutic outcomes in patients with complex pathological profiles.

## Data Availability

Not applicable.

## Abbreviations

AD: Alzheimer’s disease
ADAS-Cog: Alzheimer’s disease assessment scale–cognitive subscale
Aβ: Amyloid β
APP: Amyloid precursor protein
BBB: Blood-brain barrier
CBD: Corticobasal degeneration
CDR-SB: Clinical dementia rating–sum of boxes
CSF: Cerebrospinal fluid
CTE: Chronic traumatic encephalopathy
DAT-SPECT: Dopamine transporter single photon emission computed tomography
DLB: Dementia with Lewy bodies
GFAP: Glial fibrillary acidic protein
GWAS: Genome-wide association study
iPSC: Induced pluripotent stem cell
iRBD: Idiopathic rapid eye movement sleep behavior disorder
ISF: Interstitial fluid
LBs: Lewy bodies
LNs: Lewy neurites
MAO-B: Monoamine oxidase B
MAPT: Microtubule associated protein tau
MCI: Mild cognitive impairment
MDS-UPDRS: Movement disorder society unified Parkinson’s disease rating scale
MSA: Multiple system atrophy
MTBR: Microtubule associated protein tau
NAC: Non β-amyloid component
NFL: Neurofilament light
NFT: Neurofibrillary tangle
PD: Parkinson’s disease
PET: Positron emission tomography
PFF: Preformed fibrils
PHFs: Paired helical filaments
PDD: Parkinson’s disease with dementia
PSP: Progressive supranuclear palsy
PSPRS: Progressive supranuclear palsy rating scale
SAA: Seeding amplification assays
scFv: Single-chain antibody
TBI: Traumatic brain injury
TOMA: Tau oligomer-specific monoclonal antibody
UMSARS: Unified multiple system atrophy rating scale
